# Additive Manufacturing of Organic Electrochemical Transistors: Methods, Device Architectures, and Emerging Applications

**DOI:** 10.1002/smll.202410499

**Published:** 2025-02-13

**Authors:** Roberto Granelli, Zsolt M. Kovács‐Vajna, Fabrizio Torricelli

**Affiliations:** ^1^ Department of Information Engineering University of Brescia via Branze 38 Brescia 25123 Italy

**Keywords:** additive manufacturing, bioelectronics, fully‐printed OECTs, neuromorphics, organic electrochemical transistors

## Abstract

Organic electrochemical transistors (OECTs) are key devices in a large set of application fields including bioelectronics, neuromorphics, sensing, and flexible electronics. This review explores the advancements in additive manufacturing techniques accounting for printing technologies, device architectures, and emerging applications. The promising applications of printed OECTs, ranging from biochemical sensors to neuromorphic computing are examined, showcasing their versatility. Despite significant advancements, ongoing challenges persist, such as material‐related issues, inconsistencies in film homogeneity, and the scalability of integration processes. This review identifies these critical obstacles and offers targeted solutions and future research directions aimed at enhancing the performance and reliability of fully‐printed OECTs. By addressing these challenges, the aim of this study is to facilitate the development of next‐generation OECTs that can meet the demands of emerging applications in sustainable and intelligent electronic and bioelectronic systems.

## Graphical abstract

Organic electrochemical transistors (OECTs) are essential for bioelectronics, neuromorphics, and flexible electronics. This review examines additive manufacturing advances for OECTs, covering printing techniques, device architectures, and applications including biochemical sensing, neuromorphic, green bio‐electronics, self‐healable, and 4D electronics. Key challenges such as material limitations and integration scalability are discussed, with insights to improve OECT performance and support next‐generation, sustainable, and intelligent electronics and bioelectronics.

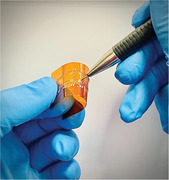

## 1 Introduction

Organic electrochemical transistors (OECTs) have emerged as key devices in the fields of bioelectronics, neuromorphics, sensing, and flexible/stretchable electronics. OECTs provide stable operation in liquid environments, high transconductance, low voltage operation, and material versatility. Their high transconductance arises from the large volumetric capacitance, which leads to efficient voltage‐to‐current transduction. This is intrinsically due to the ionic‐electronic interactions occurring throughout the entire bulk of the channel. Typically, the OECT channel is composed of soft, π‐conjugated polymers or small molecules that act as organic mixed ionic and electronic conductors (OMIECs).^[^ ^1^ ^]^ The simplicity and adaptability of OECT structures make them highly suitable for a wide range of applications, including bioelectronics, chemical sensing, and neuromorphic computing. For instance, OECTs have been successfully used in intracranial implants, in vivo neuromorphic signal acquisition, and wireless transmission,^[^ ^2^ ^]^ and allow the development of artificial neurons for neuromorphic sensing and biointerfaces.^[^ ^1^, ^3^, ^4^, ^5^, ^6^, ^7^ ^]^ Furthermore, their exceptional sensitivity to ion concentrations, coupled with their impressive efficacy as transducers for enhancing human electrophysiological signals and inherent biocompatibility, opens the door to a wide range of applications. For instance, they can monitor cellular barrier integrity in real‐time, allowing for drug screening without the need for optical labels.^[^ ^8^, ^9^ ^]^ When integrated with oxidase enzymes, they have also been used to accurately detect small changes in glucose^[^ ^10^ ^]^ and cholesterol^[^ ^11^ ^]^ levels in various media, including saliva^[^ ^12^ ^]^ and sweat.^[^ ^13^ ^]^ These OECT‐based sensors are widely used in preventive healthcare and monitoring by providing real‐time measurements of electrophysiological signals, including applications in electroencephalography (EEG),^[^ ^14^ ^]^ electromyography (EMG),^[^ ^15^ ^]^ and electrocardiography (ECG).^[^ ^16^ ^]^ Additionally, they are employed for detecting biomarkers that can predict diseases such as cancer and seizures.^[^ ^17^ ^]^ In the realm of logic circuitry, OECTs have been developed for both unipolar and complementary logic gates,^[^ ^18^, ^19^, ^20^, ^21^, ^22^, ^23^, ^24^, ^25^ ^]^ as well as multi‐value logic circuits,^[^ ^26^ ^]^ including ternary logic circuits with antiambipolar small molecule‐based materials.^[^ ^27^ ^]^ Notably, up to half a million devices have been integrated on a single substrate.^[^ ^2^ ^]^ More recently, Kim et al. achieved a breakthrough by producing up to 7.2 million monolithically stacked vertical OECTs per cm^2^ for complementary vertical logic gates.^[^ ^28^ ^]^

Given the diversity and significance of these applications, there is an increasing demand for rapid prototyping and fabrication techniques that can accommodate various substrates, geometries, architectures, and materials for OECT manufacturing. Photolithography is one of the primary fabrication methods used for OECTs, particularly for channel and source‐drain structures. This process requires the deposition of single or multiple photoresist layers by using spin coating. Spin coating, despite providing good repeatability across a variety of materials,^[^ ^29^, ^30^ ^]^ also has inherent drawbacks. The entire surface of the substrate is coated with the material, requiring additional structuring steps to define the specific areas needed for device architecture. Additionally, this approach requires organic solvents, such as acetone or photoresist developers, that can significantly degrade or delaminate the OMIEC channel. To circumnavigate these issues, parylene‐C is commonly used to protect the integrity and functionality of the organic polymer during fabrication with conventional solvents.^[^ ^31^, ^32^, ^33^, ^34^ ^]^ However, this solution introduces additional challenges. The process involves multiple applications of parylene vapor coatings and the manual removal of sacrificial parylene layers, significantly increasing both the cost and complexity of fabrication. Parylene‐C is typically deposited via chemical vapor deposition (CVD) and structured through photolithography and dry etching. In this process, two layers of parylene‐C are deposited. The OMIEC is spin‐coated, and the OECT channel is patterned by mechanically peeling off the second parylene‐C layer.^[^ ^19^, ^35^, ^36^ ^]^ These extra steps hinder the efficient integration of OECTs with various architectures, making large‐scale production more difficult. Additionally, wet‐etching methods lead to considerable material waste, with up to 90% of the material being lost during the process, either spun off during coating or removed in subsequent steps.

To address these challenges, non‐lithographic processes have gained significant attention in recent years. These emerging technologies allow the deposition of conductive, insulating, and functional polymer semiconducting inks through various additive manufacturing techniques, enabling the fabrication of large‐scale, low‐cost electronic systems, and devices. Some of the most promising printing methods include screen printing,^[^ ^37^, ^38^ ^]^ inkjet printing,^[^ ^39^, ^40^ ^]^ aerosol‐jet printing,^[^ ^41^, ^42^ ^]^ dispensing,^[^ ^43^, ^44^ ^]^ gravure printing,^[^ ^45^, ^46^ ^]^ transfer printing,^[^ ^47^, ^48^ ^]^ and electrodynamic inkjet (e‐jet) printing,^[^ ^49^, ^50^ ^]^ These techniques offer an alternative to photolithography by providing direct patterning without the need for solvents and material etching. The integration of organic electrochemical transistors (OECTs) with printing technologies, combined with features like stretchability, flexibility, and seamless integration, opens new pathways for electronic and bioelectronic applications. These include sensors,^[^ ^51^, ^52^, ^53^ ^]^ logic circuits,^[^ ^20^, ^54^ ^]^ active matrix,^[^ ^46^, ^55^ ^]^ and healthcare diagnostic and prevention.^[^ ^8^, ^56^, ^57^ ^]^ Emerging additive manufacturing (AM) technologies can offer precise control over parameters such as electrode composition, geometry, channel volume, topography, structure, and electrolyte properties, enabling on‐demand OECT fabrication. Therefore, this approach provides significant advantages for optimizing materials and improving device engineering. For example, printable OMIECs with excellent mechanical properties—such as high Young's modulus and cracking strain,^[^ ^58^, ^59^, ^60^ ^]^ can be fabricated alongside materials with superior electrochemical characteristics, including ion mobility and resistivity^[^ ^61^, ^62^, ^63^ ^]^ Some of these materials also feature self‐healing capabilities,^[^ ^62^, ^64^, ^65^ ^]^ making them ideal for flexible electronics. AM techniques further enable seamless, monolithic integration and 3D structuring,^[^ ^20^, ^24^, ^66^ ^]^ allowing devices to be printed on unconventional substrates like flowers and peppers.^[^ ^43^ ^]^ Additionally, the incorporation of ionic liquids and gels has led to the development of entirely solid‐state OECTs.^[^ ^67^, ^68^ ^]^ Moreover, AM supports the creation of partially and fully biodegradable devices, which are entirely recyclable, minimizing waste and promoting environmentally sustainable electronics.^[^ ^44^, ^65^, ^66^, ^69^, ^70^, ^71^ ^]^

This review explores the latest advances in AM technologies with their potential to revolutionize the development of OECTs for a range of bioelectronic and electronic applications. Compared to conventional microfabrication methods, AM offers greater flexibility, scalability, and cost efficiency while reducing environmental impact. These advantages are key to driving innovation in the production of next‐generation OECTs. The paper is structured to first provide an overview of the fundamentals of OECTs, including various architectures and operating principles. This foundational section introduces the core characteristics of OECTs and the factors that influence their performance, setting the stage for a deeper exploration of how AM technologies can address the challenges of traditional fabrication methods. A central focus is on OECT printing technologies, where various AM techniques are examined for their roles in device fabrication and integration. Recent advances in printable OMIECs are discussed, highlighting their mechanical and electrochemical properties, such as high ion mobility and self‐healing capabilities. By integrating these advanced materials with AM processes, promising new strategies emerge to enhance the performance of OECTs. Emerging applications of AM‐fabricated OECTs are presented in fields like bioelectronics, healthcare diagnostics, and environmental monitoring. The ability to integrate OECTs into stretchable and flexible devices opens new possibilities for innovative electronic systems that can directly interface with biological environments. The review emphasizes the role of AM technologies in enabling the production of biodegradable and recyclable OECTs, showing that these devices can be key contributors to sustainable electronics and bioelectronics. Finally, we present a forward‐looking perspective on overcoming current limitations in OECT fabrication through AM. The conclusion highlights the potential of AM techniques to shape the future of large‐scale, low‐cost electronics manufacturing, fostering rapid innovation, and expanding the range of applications within the field.

## 2 Fundamentals of Organic Electrochemical Transistors

### 2.1 OECT Structures

**Figure 1:**
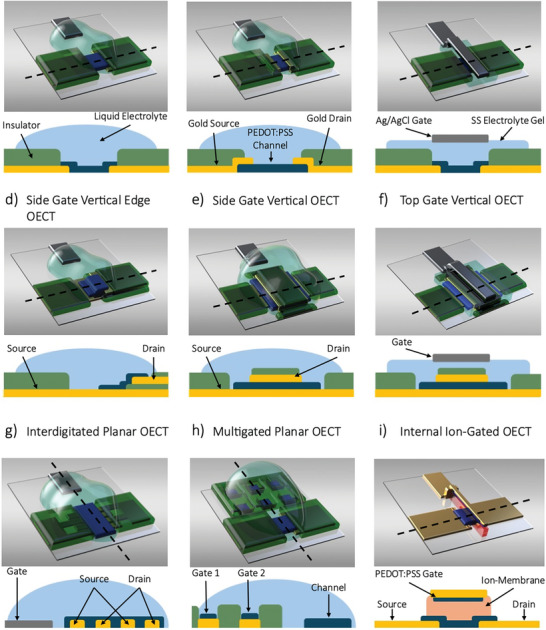
Basic structures and cross‐sections of organic electrochemical transistors (OECTs). Substrate is transparent in the 3D views and it is not shown in the cross‐section views, the source, drain electrodes are shown in yellow, polarizable gate electrode is shown in yellow, the non‐polarizable gate electrode is shown in grey, the OMIEC used for channel material and gate electrode is shown in blue, the electronic and ionic insulator is shown in green, the ion membrane is shown in orange, the electrolyte is semi‐transparent cyan in the 3D views and it is full cyan in the cross‐section views. a) Side‐gate bottom contacts planar OECT. b) Side‐gate top contacts planar OECT. c) Top‐gate planar OECT. d) Vertical edge‐channel OECT with side gate. e) Vertical OECT with side gate. f) Vertical OECT with top gate. g) Interdigitated planar OECT. h) Multi‐gated planar OECT. i) Internal ion‐gated transistor (IGT).

OECTs can be designed with various device architectures, depending on factors such as the position of the gate electrode relative to the semiconductor channel, the arrangement of the source and drain electrodes, and the number of electrodes. The most commonly used design is the side‐gated planar OECT (**Figure** **1**a), which features a bottom‐contacted channel. This configuration is relatively simple to fabricate and is particularly advantageous for biological applications because the maximum channel area is exposed to the electrolyte, allowing for bio‐functionalization and high‐performance bio‐detection applications.^[^ ^72^, ^73^, ^74^ ^]^ To enhance OECT performance in other areas, such as improving the amplification of electrophysiological signals, alternative architectures have been explored.^[^ ^2^, ^75^, ^76^ ^]^ For example, Paudel et al. analyzed a planar OECT structure with a top‐contact source and drain electrodes. When compared to the conventional bottom‐contacted OECT, the top‐contact design (Figure 1b), demonstrated lower contact resistance due to a larger contact area between the channel and electrodes. As a result, the current flow increased, and the response time constant (τ) improved. In this design, cations along the transistor channel undergo a lateral drift caused by the lateral electric field created by the drain potential, causing them to accumulate near the drain electrode.^[^ ^77^ ^]^ Recent advancements in solid‐state electrolytes have further expanded the scope of OECT applications. First Ersman et al. and after Boda et al. introduced a fully printed top‐gated planar OECT architecture (Figure 1c),^[^ ^20^, ^38^ ^]^ aiming to enable the monolithic integration of OECT‐based logic circuits with displays. This structure, featuring an electrolyte sandwiched between the channel and gate electrode, is similar to the pixel‐electrolyte‐counter electrode structure found in electrochromic displays and active matrices.^[^ ^20^, ^38^, ^78^, ^79^ ^]^ Recent advancements in OECT architectures have focused on vertically stacked structures involving the source‐channel‐drain elements. The first of these, developed by Donahue et al., features a lower gold electrode partially covered by a parylene‐C layer, over which a second gold electrode is thermally evaporated, forming a ladder‐like structure onto which the channel is deposited (Figure 1d). This vertical design significantly reduces the channel length, achieving dimensions as small as 450 nm.^[^ ^80^ ^]^ According to the relationship *g_m_* ∝*Wd*/*L*, where *W* is the channel width, *d* the thickness and *L* is the length, this configuration results in exceptionally high transconductance (*g_m_*), with values reaching 25 mS. Similar structures developed by Brodský et al. demonstrated even higher transconductance values of 68 mS with a channel length of 280 nm.^[^ ^81^ ^]^ Building on these developments, Huang et al. recently created both p‐type and n‐type vertical OECTs for complementary circuit applications. In their design, they eliminated the insulator layer that previously defined the channel length, instead developing a cross‐structure where the source and drain electrodes are separated solely by the polymer channel (Figure 1e). This innovation further reduced the channel length to ≈100 nm, enabling devices with maximum transconductance as high as 384 mS and an on/off current ratio exceeding 10^6^.^[^ ^21^ ^]^ Recently, Moon et al. introduced the first fully vertical OECT (Figure 1f), integrating the gate electrode into a vertical OECT structure using a solid‐state electrolyte.^[^ ^24^ ^]^ This design enabled the implementation of unipolar logic gates such as NOT, NAND, and NOR. Inspired by solid‐state silicon technology, further advancements included the interdigitation of source and drain electrodes, as demonstrated by AlChamaa using inkjet technology, which allowed for tuning the current by altering the *W*/*L* ratio (Figure 1g).^[^ ^39^ ^]^ Another innovative architecture was developed by Koutsouras et al. demonstrating that multi‐gated OECTs can function as iontronic multiplexers, utilizing space‐time gating actions to enable the simultaneous control and processing of multiple signals (Figure 1h).^[^ ^82^, ^83^ ^]^ One of the latest advancements in OECT architectures is the Internal Ion‐Gated organic electrochemical Transistor (IGT), fabricated by Spyropoulos et al. via photolithography. This design incorporates an ion‐membrane directly between the gate electrode and the channel (Figure 1i).^[^ ^18^ ^]^ The key feature of IGTs is the channel's rich concentration of embedded mobile ions, enabling a self‐(de)doping process that eliminates the need for ion exchange with an external electrolyte. Building on this, Cea et al. later developed enhancement‐mode IGTs with a vertical structure for real‐time in vivo electrophysiological processing and stand‐alone operation. These devices, with a density of 155 000 per cm^−2^, also allow for wireless signal transmission.^[^ ^2^, ^19^ ^]^

The various structure of OECTs comprises five components: i) substrate, ii) source, and drain electrodes, iii) channel, iv) gate electrode, and v) electrolyte. Regarding substrates, glass is commonly used, but its rigidity renders it unsuitable for applications in flexible or stretchable electronics and bioelectronics, particularly in scenarios where devices need to be attached to or implanted within the human body. Other critical substrate properties include flatness, surface roughness, and wettability. To modify the surface tension of the substrate, various treatments can be employed, such as UV‐Ozone cleaning,^[^ ^44^, ^84^ ^]^ self‐assembled monolayers (SAMs), or even the direct incorporation of surface modifiers into the ink.^[^ ^85^ ^]^ The source and drain electrodes in OECTs are typically made of gold, due to its low electroactivity at low oxidative potentials and its ability to form excellent ohmic contacts with typical OMIECs used for channel materials. Gold electrodes are commonly deposited via sputtering or thermal evaporation,^[^ ^21^, ^31^, ^86^, ^87^, ^88^ ^]^ but recent advancements in printing technologies allow for the deposition of gold‐based nanoparticle inks. Techniques such as inkjet and aerosol‐jet printing allow the printing low‐viscosity inks (<10 cPs), expanding fabrication possibilities.^[^ ^40^, ^89^, ^90^, ^91^, ^92^ ^]^ In addition to gold, silver electrodes and interconnections are also widely used in printed OECTs. Silver is available as nanoparticle‐based inks dispersed in aqueous solutions or as high‐viscosity pastes, suitable for methods like dispensing, gravure, and screen printing.^[^ ^37^, ^39^, ^93^, ^94^ ^]^ Silver is a cost‐effective alternative to gold, often favored for its lower curing temperatures. In OECTs, silver is commonly used as a gate material, where it is directly exposed to the electrolyte, acting as a non‐polarizable electrode. However, when silver is used for source and drain electrodes or interconnections, it typically requires encapsulation with an ionic insulator. This encapsulation prevents undesirable side reactions that could otherwise affect the device's stability and performance. Carbon‐based conductive inks are increasingly preferred in OECT applications due to their high stability, low electroactivity, and superior biocompatibility.^[^ ^22^, ^43^, ^44^, ^54^, ^66^, ^95^, ^96^, ^97^, ^98^, ^99^ ^]^ These properties make them ideal for a wide range of applications, particularly in bioelectronics and sensing, where material compatibility and long‐term stability are crucial. Additionally, carbon inks are available in both liquid and paste forms, making them compatible with virtually all printing methods. Their viscosities can range from just a few centipoise (cP) for inkjet printing to several hundred thousand cP for screen printing.^[^ ^37^, ^69^, ^97^ ^]^ Liquid carbon inks typically consist of carbon particles dispersed in water or solvents like N‐methyl‐2‐pyrrolidone (NMP),^[^ ^100^ ^]^ whereas pastes are formulated by mixing carbon or graphite powder with various binders and surfactants, including mineral oil, silicone oil, beeswax, and kerosene.^[^ ^101^, ^102^ ^]^ The curing temperature of carbon inks is often compatible with biodegradable substrates such as cellulose diacetate,^[^ ^44^ ^]^ polylactic acid (PLA),^[^ ^69^ ^]^ poly(lactic‐co‐glycolic acid) (PLGA),^[^ ^16^, ^103^ ^]^ and paper sheets.^[^ ^71^ ^]^ Carbon‐based inks are also frequently utilized as gate electrodes in OECT sensors for the detection of glucose and fructose.^[^ ^104^, ^105^, ^106^ ^]^ Next, the main component of OECTs is the OMIEC channel. The most widely studied and used OMIEC is the conductive polymer poly(3,4‐ethylenedioxythiophene) complexed with poly(styrene sulfonate) (PEDOT:PSS). PEDOT:PSS results in depletion‐mode (normally‐ON) OECTs and it is favored for several reasons: it is stable in aqueous environments, can be processed using various fabrication methods at low temperatures (typically 140 °C or below), combines large hole mobility (typically 1–2 cm^2^ V^−1^ s^−1^) with significant volumetric capacitance (> 40 F cm^−3^), and is commercially available. Alongside the increasing use of OECTs, the development of OMIECs has also expanded significantly. This has led to a focus on undoped polymers suitable for enhancement‐mode (normally‐OFF) OECTs. One prominent example is poly(3‐hexylthiophene) (P3HT), the most extensively studied p‐type homopolymer, recognized for its effectiveness in charge transport and structural formation in both solution and thin‐film states. The key approach for developing polar semiconducting polymers involves attaching polar side chains to the backbone of π‐conjugated polymers. This modification facilitates ion adsorption and transport throughout the polymer bulk while preserving the charge transport capabilities characteristic of conjugated polymers. A notable example of such a copolymer is poly(2‐(3,3′‐bis(2‐(2‐methoxy‐ethoxy)ethoxy)‐[2,2′‐bithiophene]5‐yl)thieno[3,2‐b]thiophene) (p(g2T‐TT)).^[^ ^25^, ^107^, ^108^, ^109^ ^]^ In the case of n‐type OECTs, poly(benzimidazo benzophenanthroline) (BBL) is widely used as the electroactive channel material. BBL belongs to the ladder‐type polymer class, distinguished by its high rigidity and planarity in the π‐conjugated polymer backbone. This structural feature enhances the delocalization of charge carriers, facilitating intramolecular transfer, and resulting in high polaron mobility along the ladder‐like chains. Other n‐type materials are often based on naphthalene diimide (NDI) derivatives and small molecules, which show very large volumetric capacitance. **Table** **1** presents the volumetric capacitance and the mobility of OMIEC materials widely used for OECTs.

**Table 1:** Transport type, volumetric capacitance (C*), and electronic mobility (*µ*) of widely used OMIECs. The µC* factor captures the mixed ionic‐electronic transport properties of the OECT channel material.

Material	Transport type	C* [F cm^−3^ ]	μ [cm^2^ V^−1^ s^−1^]	Refs.
p(g2T‐TT)	p	241	0.94	[110]
p(g2T‐T)	p	220	0.28	[111]
PEDOT:TOS	p	136	0.93	[112]
PEDOT:PSS + EG	p	39	1.9	[113]
PEDOT:PSTFSILi100	p	26	0.23	[114]
PTHS + EG	p	124	0.0013	[115]
p(gBDT‐g2T)	p	77	0.018	[111]
PEDOT:DS + EG	p	65	0.0064	[116]
p(gNDI‐g2T)	p	397	0.00031	[117]
PEDOT:PMATFSILi80	p	27	0.0024	[114]
BBL	n	731	0.00214	[118]
P4gNDI	n	219	0.000007	[119]
P4gNDTI	n	167	0.00142	[119]
PBBTL:BBL	n	168	0.0081	[120]
P90	n	91.6	0.0000098	[121]
p(gNDI‐gT2)	n	221	0.00022	[122]
P(gTDPP2FT)	n	156	0.27	[123]
P(DPP‐TDP)	n	68	0.11	[124]
gAIID‐T	n	43	0.002	[125]
P(g7NC2N)	n	180	0.002	[126]

In OECTs, insulating materials play a crucial role by preventing direct contact between the source and drain electrodes and the electrolyte. Direct contact of the electrolyte with the electrodes increases the parasitic capacitances and leakage currents, adversely affecting the electrical performance of the OECTs. This can result in a reduced on/off current ratio, increased turn‐off current, and higher power consumption. Dielectric inks are available for all printing methods and come in liquid or paste forms with varying viscosities and particle sizes. These inks are typically composed of non‐conductive hydrophobic plastic polymers or sol–gel metal oxides, making them some of the most versatile materials in the fabrication process. They enable manufacturing at room temperature with methods such as UV curing or low‐temperature annealing. Examples include polydimethylsiloxane (PDMS) and optical adhesives.^[^ ^127^, ^128^, ^129^, ^130^, ^131^ ^]^ Another key component of OECT structures is the electrolyte, which influences the ionic and ionic‐electronic characteristics of the devices. In bioelectronics, the electrolytes include biofluids such as blood,^[^ ^132^ ^]^ sweat,^[^ ^74^ ^]^ saliva,^[^ ^57^ ^]^ and even plant sap.^[^ ^133^ ^]^ Recent advancements in logic circuits and frequency operations have led to the development of high‐performance solid‐state OECT technology with monolithic integration. For instance, various types of gels—including hydrogels,^[^ ^44^, ^134^, ^135^ ^]^ euctogels,^[^ ^136^ ^]^ and glycerol gels have been developed,^[^ ^137^ ^]^ often based on polymer matrices such as polyvinyl alcohol (PVA),^[^ ^68^, ^93^, ^138^, ^139^ ^]^ poly(sodium 4styrene sulfonate) (PSS:Na)^[^ ^43^, ^44^ ^]^ and organic celluloses like sodium carboxymethyl cellulose (CMC:Na).^[^ ^140^, ^141^ ^]^ The characteristics of solid‐state electrolytes can change depending on the ionic components used, which may be water‐based or ionic liquid‐based. These solid‐state electrolytes enhance the stability and integration of OECTs. Additionally, introducing a solid layer above the channel facilitates the printing of a top gate electrode, thereby promoting miniaturization and the vertical structuring of OECTs.^[^ ^38^, ^66^, ^95^, ^97^ ^]^ Using non‐lithographic printing processes allows for the deposition of non‐polarizable gate electrodes, employing materials such as silver/silver chloride (Ag/AgCl) ink paste or silver‐based inks.^[^ ^24^ ^]^

### 2.2 Operating Principle of OECTs

**Figure 2:**
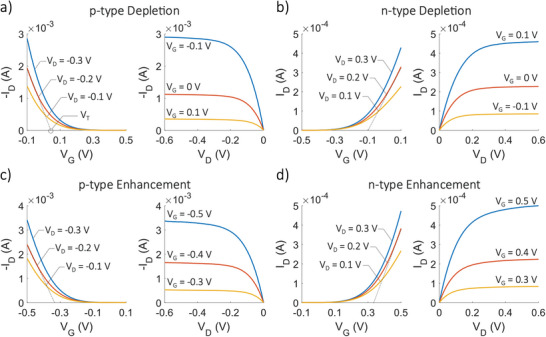
Representative electrical characteristics of unipolar OECTs. Transfer (I_D_‐V_G_) and output (I_D_‐V_D_) characteristics at various *V*_G_ and *V*_D_ of a) p‐type depletion mode OECTs, b) n‐type depletion mode OECTs, c) p‐type enhancement mode OECTs, and d) n‐type enhancement mode OECTs.

The operating principle of OECTs is based on the modulation of the channel conductivity through ionic and electronic interactions. When a voltage is applied to the gate electrode, ions from the electrolyte penetrate the organic semiconductor channel. These ions modulate the channel conductivity by doping or dedoping the material, effectively controlling the current flow between the source and drain electrodes. **Figure** **2** presents typical electrical characteristics of OECTs, which can be described using the model developed by Bernards and Malliaras.^[^ ^142^ ^]^ The drain current (I_D_) of an OECT operating in the linear region (V_G_−V_S_ > V_T_ and V_G_−V_D_ > V_T_) and in the saturation region of operation can be expressed as:

(1) $$I_{D}=\frac{W}{L}d\mu C^{*}\left(V_{T}-V_{G}+\frac{V_{D}}{2}\right)V_{D}$$

(2) $$I_{D}=\frac{W}{L}d\mu C^{*}{\left(V_{T}-V_{G}\right)}^{2}$$

where W and L are the channel width and length, respectively, *d* is the channel thickness, μ is the electronic mobility in the channel, C* is the volumetric capacitance of the channel, and *V_T_* is the threshold voltage. The transient response of OECTs depends on both the electronic and ionic charge dynamics. Assuming a uniform doping (or de‐doping) process in the channel, Bernard's model predicts that the *I*_D_ response to a square *V*_G_ pulse follows an exponential time dependence, and reads:

(3) $$I_{D}\left(V_{G},t\right)=I_{SS}\left(V_{G}\right)+\Delta I_{SS}\left(1-f\frac{\tau_{e}}{\tau_{i}}\right)e^{\left(-\frac{t}{\tau_{i}}\right)}$$

where *I_SS_* (*V_G_*) is the steady‐state current at the applied gate voltage *V_G_*, Δ*I_SS_* is the difference between the initial and final steady‐state currents, *f* is a weighting factor corresponding to the *I_G_* contribution to *I_D_*. τ_*e*_ is the electronic time constant in the channel, and τ_*i*_ corresponds to the ionic time constant obtained from the equivalent ionic‐electronic circuit: *R_s_* (*R_p_* ||*C_ch_*), where *R_p_* and *C_ch_* is the parallel resistance and capacitance of the polymeric channel, respectively, and *R_s_* the ionic resistance of the electrolyte. The channel capacitance *C_ch_* = C*/*WLd* can be calculated from the volumetric ionic‐electronic capacitance and the channel dimensions. The ionic resistance *R_s_* is proportional to $1/\sqrt{WL}$.^[^ ^14^, ^143^, ^144^ ^]^ The Bernard's model was the first attempt to predict the transient response of OECTs, and more recent models have improved predictive capabilities. Friedlein et al. introduced a model that considers an exponential distribution of the density of states. This model accounts for the influence of charge carrier density on mobility, addressing the assumption of constant mobility in Bernard's model. By setting *f*  =  1/2 they established the condition for operating at the boundary between the monotonic and spike‐recovery regimes.^[^ ^145^ ^]^ Further descriptions of the OECT operating principles can be found in the reviews by Ohayon et al., Zhao et al., and Colucci et al.^[^ ^146^, ^147^, ^148^ ^]^

Depending on the intrinsic doping state of the channel material, unipolar OECTs can operate as depletion‐mode or enhancement‐mode transistors. Representative transfer (*I*_D_‐*V*_G_) and output (*I*_D_‐*V*_D_) electrical characteristics of p‐type and n‐type depletion mode OECTs are shown in Figures 2a,b, respectively. In depletion mode, the channel is made of a doped semiconducting material. When a positive gate voltage (*V*_G_) is applied, ions from the electrolyte penetrate the channel, neutralizing the dopant charges and reducing the number of charge carriers (e.g., holes or electrons), leading to a decrease in channel conductivity. As V_G_ increases, the carrier concentration continues to decrease, eventually turning the device OFF. When *V*_G_ is reversed, the ions drift out of the channel, restoring the carrier concentration and turning the device back ON. For instance, depletion‐mode p‐type OECTs can be obtained using PEDOT:PSS, where the negative charges in the polyelectrolyte PSS give rise to a large concentration of holes in the PEDOT phase. Analogously, depletion‐mode n‐type OECTs can be obtained with BBL:PEI, where the positive charges in the polyethyleneimine (PEI), an amine‐based polyelectrolyte, give rise to a large concentration of electrons in the BBL phase. In this configuration, the negatively charged BBL chains align preferentially parallel to the substrate and are electrostatically compensated by the long, positively charged PEI chains, which also align parallel to the substrate. This alignment promotes anisotropic in‐plane conductivity, similar to the behavior observed in PEDOT:PSS. Notably, BBL:PEI forms an ohmic contact with various electrodes, exhibiting compatibility with work functions ranging from 5.1 eV (PEDOT:PSS) to 2.8 eV (Ca/Al).^[^ ^63^ ^]^

Although PEDOT and its derivatives remain the most widely used p‐type materials for OECT channels, research into p‐type materials for enhancement‐mode OECTs has gained significant momentum in recent years. Enhancement‐mode OECTs, unlike their depletion‐mode counterparts, are normally OFF when no gate voltage is applied. This behavior stems from the fact that the channel material is undoped or minimally doped in its default state, resulting in low or negligible conductivity. Representative transfer electrical characteristics of p‐type depletion mode OECTs are shown in Figure 2c. The operating principle of enhancement‐mode OECTs relies on applying a positive *V*_G_ to turn the device ON. When a positive gate voltage is applied, cations from the electrolyte diffuse into the channel, doping the semiconducting material and increasing the concentration of charge carriers (such as holes in p‐type materials). This process enhances the conductivity of the channel, allowing current to flow between the source and drain. When *V*_G_ is removed or reversed, the ions leave the channel, reducing the carrier concentration and turning the device OFF. This on/off switching mechanism is particularly beneficial for low‐power and energy‐efficient applications, as the device consumes little to no power when in the OFF state. The development of new p‐type materials for enhancement‐mode OECTs has focused on optimizing several key properties, including ionic‐electronic coupling, stability in aqueous environments, and the ability to achieve high current modulation. Materials such as thiophene‐based polymers, diketopyrrolopyrrole (DPP) derivatives, and certain conjugated polyelectrolytes have demonstrated potential for use in these devices. Moreover, robust methods for the synthesis of regioregular polymers with controlled molar mass and functionalization of the terminal chain have made thiophene and its derivatives promising materials for p‐type OECT channels.^[^ ^149^ ^]^ Thiophene is an organosulfur heterocycle whose aromatic nature makes it amenable to various substitution reactions, leading to the development of environmentally, chemically, and thermally stable polythiophenes. Among these, regioregular poly(3‐hexylthiophene‐2,5‐diyl) (P3HT) is particularly notable.^[^ ^150^ ^]^ Its ease of processing compared to non‐functionalized polythiophenes has made it an attractive candidate for OECTs.^[^ ^26^, ^151^ ^]^ Nielsen and colleagues synthesized a series of thiophene‐based precursors, including those incorporating triethylene glycol (TEG) side chains. TEG, with its electron‐rich conjugated system and plasticizer side chains, enhances both ionic and electronic transport throughout the polymer film, leading to improved performance in accumulation‐mode OECTs.^[^ ^111^ ^]^ Among these materials, poly(2‐(3,3′‐bis(2‐(2‐(2‐methoxyethoxy)ethoxy)ethoxy)‐[2,2′‐bithiophen]‐5)yl thiophene) (p(g2T‐T)) showed promising results, and its glycolated version, p(g2T‐TT), emerged as the top performer.^[^ ^110^, ^152^ ^]^

In recent years, significant progress has been made in exploring n‐type materials for enhancement‐mode OECTs.^[^ ^117^, ^153^, ^154^ ^]^ Representative transfer electrical characteristics of n‐type enhancement mode OECTs are shown in Figure 2d. Side‐chain‐free polymers, such as BBL and its derivatives, have been the most commonly used in this category. For instance, Huang et al. reported the development of a highly conductive n‐type polymer called PBFDO,^[^ ^154^ ^]^ marking a significant advancement in the performance of side‐chain‐free materials. Other families of n‐type studied materials include the naphthalene diimide (NDI) derivatives, the bithiophene imide dimer (BTI) derivatives, the diketopyrrolopyrrole (DPP) derivatives, and the isoindigo (IID) derivatives.

**Figure 3:**
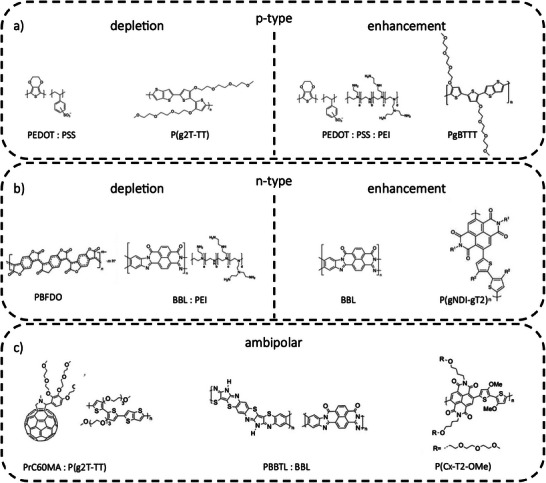
Chemical structures of widely used a) p‐type materials, b) n‐type materials, and c) ambipolar blends and materials used for OECTs fabrication.

Recent advancements in OECT research are also focusing on the use of crystalline materials for the development of OECT channels. Among these, small molecules have garnered attention due to their well‐defined and consistent structures, which exhibit minimal variation across production batches. This consistency enables reliable and reproducible device performance, a critical factor for scalable applications. The monodisperse nature of small molecules establishes clear structure‐property relationships, facilitating the optimization of structural ordering and the enhancement of mixed ionic and electronic charge transport. Molecular design and iterative synthesis can thus be employed to achieve ideal configurations for superior device performance. Recent studies on OECT channel materials highlight the growing interest in semiconducting small molecules due to their superior electronic mobility (20–40 cm^2^ V^−1^ s^−1^), which is notably higher than that of polymeric counterparts. A milestone in this area was achieved by Ginger et al.,^[^ ^155^ ^]^ who reported the first n‐type small‐molecule OMIEC, a fullerene derivative with hydrophilic OEG side chains, known as C60‐TEG. This demonstrated the potential of small‐molecule semiconductors as promising candidates for the development of ultra‐high‐performance OECTs.^[^ ^156^ ^]^ Small molecule n‐type organic semiconductors have already demonstrated outstanding results in organic field‐effect transistors (OFETs).^[^ ^157^ ^]^ In theory, these materials should also offer great potential for OMIEC‐based applications, including OECTs. The advantage of small molecules lies in their well‐defined structures, which exhibit minimal batch‐to‐batch variation, leading to more reliable and reproducible device performance. Their monodisperse nature allows for precise structure‐property relationships, enabling targeted molecular design and synthetic adjustments to optimize structural ordering and enhance mixed ionic and electronic charge transport.^[^ ^156^, ^158^, ^159^ ^]^ The family of small molecules can be categorized into fullerene‐based, PDI‐ and NDI‐based, isatin‐based, and DPP‐based materials. **Figure** **3** illustrates some of the polymers and molecules commonly used in OECT fabrication processes.

Beyond small molecules, other innovative material categories are emerging. These include 2D polymers and 2D metal‐organic frameworks (MOFs), which feature ordered nanoporous structures.^[^ ^160^, ^161^ ^]^ Such materials are particularly well‐suited for OECT applications: their in‐plane extended π‐conjugated networks support efficient charge transport, while vertical nanopores provide optimal pathways for ion penetration. Despite their potential, research on these advanced materials remains in its early stages, with only a limited number of studies exploring their use in OECTs. Examples of advancements in 2D materials for OECT development include the 2D ordered polymer TIIP, which has been successfully employed to fabricate high‐performance p‐type OECTs.^[^ ^160^ ^]^ 2D conjugated cMOFs, such as Cu₃(HHTP)₂ enable a reproducible layer‐by‐layer growth method and serve as the channel material for ambipolar MOF‐based ECTs.^[^ ^161^ ^]^ Both material classes exhibit exceptional properties critical for advanced OECT performance: high charge carrier mobility, enhanced ion permeability, robust long‐term chemical stability, and rapid response times. These characteristics position them as promising candidates for next‐generation OECT technologies.

**Figure 4:**
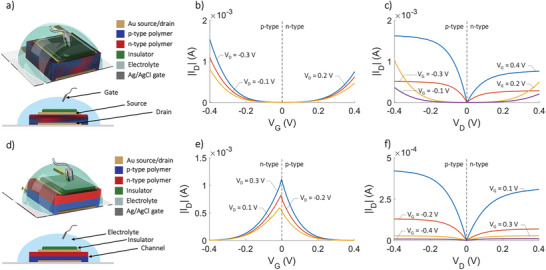
Schematic structures, cross sections, and representative electrical characteristics of ambipolar and anti‐ambipolar vertical OECTs. a) Ambipolar OECT, the channel is composed of a blend of both p‐type and n‐type polymers. b) Typical transfer characteristics and c) output characteristics of ambipolar OECTs. d) Anti‐ambipolar OECT, the channel is composed of two layers, one is a p‐type polymer and the other is a n‐type polymer. e) Typical transfer characteristics and f) output characteristics of anti‐ambipolar OECTs.

A significant part of the state‐of‐art materials for OECT channel are obtained from the design principles of donor/acceptor conjugated polymers (D‐A CPs).^[^ ^162^, ^163^ ^]^ The absolute values of the energy levels for the highest occupied molecular orbitals (HOMO) and lowest unoccupied molecular orbitals (LUMO) must be compatible with those of the adjacent buffer or electrode layers. There are three principal ways to modify the frontier orbital energies: i) enlarging the π orbital systems;^[^ ^164^ ^]^ ii) incorporating planar fused aromatic ring systems such as quinoidal structures;^[^ ^165^ ^]^ and iii) incorporating alternating donor/ acceptor functional units.^[^ ^166^ ^]^ While these methods are very effective for reducing energy gaps, both options (i) and (ii) have the adverse effect of increasing HOMO energy levels. Method (iii) overcomes this problem by integrating electron‐rich and electron‐deficient molecular groups into the donor polymer layer, thereby providing a local acceptor character that affects frontier orbital energy levels and possibly also exciton lifetimes. In such copolymer systems, interactions between alternating D–A units are strong enough to cause the HOMO/LUMO gap to shrink. Through further investigation of conductive polymers and the application of innovative structures in organic electrochemical transistor design, ambipolar, and anti‐ambipolar OECTs were developed. Representative device structure and typical transfer electrical characteristics of ambipolar and antiambipolar OECTs are shown in **Figure** **4**a–f, respectively.

Ambipolar OECTs are designed to conduct both types of charge carriers—holes (p‐type) and electrons (n‐type)—in the same device channel. The channel material in ambipolar OECTs is typically a semiconducting polymer or small molecule that can support the transport of both holes and electrons, depending on the applied gate voltage. When a positive *V*_G_ is applied, the device typically operates in n‐type mode, where cations from the electrolyte enter the channel, facilitating electron transport. Conversely, when a negative *V*_G_ is applied, the device operates in p‐type mode, where anions penetrate the channel, enabling hole conduction. The ability to switch between both carrier types gives ambipolar OECTs unique versatility, allowing them to function in complex logic circuits and as sensors with the ability to detect both oxidative and reductive processes. The first ambipolar OECT material reported was p(gNDI‐gT2), in which both NDI and T2 monomers were modified with linear ethylene glycol (EG) side chains. This material demonstrated strong n‐type performance, though its p‐type properties were relatively weak. Subsequently, Ohayon et al. discovered that substituting the T2 unit in the NDI‐T2 structure with OMe or EG groups led to an increase in the HOMO levels. This modification resulted in a reduced energy gap, producing ambipolar materials, albeit at the cost of diminished n‐type performances.^[^ ^167^ ^]^ In comparing the properties of p(C_4_‐T2‐C0‐OMe) and the n‐type polymer p(C_4_‐T2) with no substituents on the T2 unit, p(C_4_‐T2‐C0 OMe) exhibits p‐type character. However, its n‐type performance drops significantly, with the µC* decreasing from 0.30 F cm^−1^ V^−1^ s^−1^ in p(C_4_‐T2) to 0.07 F cm^−1^ V^−1^ s^−1^. When the substituent on the T2 unit is changed from OMe to EG in p(C_4_‐T2‐C0‐EG), a slight recovery in n‐type transport characteristics was observed, although this reduced the p‐type performance. Additionally, the use of bulk‐heterojunction blends represents another effective strategy for developing ambipolar OECT materials. One notable example of blending materials involves the p‐type polymer p(g2T‐TT) combined with the n‐type fullerene derivative PrC_60_MA, which has a structure similar to C_60_‐TEG.^[^ ^168^ ^]^ Another approach is blending two ladder‐type polymers, such as n‐type BBL and p‐type PBBTL.^[^ ^120^ ^]^ The actual coupling between n‐type and p‐type polymers was first investigated by Uguz et al., who integrated the face‐to‐face p‐type material p(g‐2T‐TT) with the n‐type material C_6_NDI‐T to develop front‐end amplifiers.^[^ ^25^ ^]^ They successfully realized a push‐pull amplifier composed of an enhancement‐mode p‐type OECT and an n‐type OECT, achieving a small signal gain of over 30 dB. In addition, the combination of a p‐type p(g2T‐TT) OECT in enhancement mode with a p‐type depletion‐mode PEDOT:PSS OECT load resulted in overlapping output characteristics for the same input voltage. This integration enabled the development of a differential amplifier with a significant common‐mode rejection rate exceeding 60 dB, facilitating applications in the in vivo recording of neuronal signals.

The interfacing between p‐type and n‐type polymers, along with various modes of operation, has recently led to the development of antiambipolar OECTs. Antiambipolar OECTs typically have a similar structure to ambipolar devices, but the key difference lies in the channel material or material combination. In antiambipolar devices, two different semiconducting materials—one n‐type and one p‐type—are often combined in the channel or at the interface. The source and drain electrodes are connected at either end, while an electrolyte and a gate electrode modulate the ion injection into the channel. The defining feature of an antiambipolar OECT is that it exhibits a peak in conductivity at intermediate V_G_ values (Figure 4e), where both p‐type and n‐type carriers are present in the channel but do not overlap or fully conduct. Instead of maintaining high conductivity at both high positive and high negative gate voltages (as in ambipolar OECTs), the device conductivity sharply decreases as the gate voltage moves away from the intermediate region toward extreme positive or negative values. At intermediate V_G_, both holes and electrons contribute to moderate conduction, creating a peak in current. At very high or very low V_G_, either hole or electron conduction dominates, but the other carrier type is suppressed, leading to low overall conductivity. The resulting antiambipolar behavior leads to a unique “inverted V” current‐voltage curve, where the device exhibits low conductivity at both extremes of V_G_ and higher conductivity in the middle range. Antiambipolar OECTs are emerging as building blocks for ternary logic circuits^[^ ^27^ ^]^ (which can encode three logic states instead of two), as well as in neuromorphic computing^[^ ^7^ ^]^ and advanced sensing applications^[^ ^169^ ^]^ where complex switching and signal processing are required. Recently, Laswick et al. proposed a vertical p‐n bilayer OECT exhibiting anti‐ambipolar OFF‐ON‐OFF states. Thus, by interfacing enhancement‐mode n‐type ladder polymer, BBL, and the depletion‐mode p‐type PEDOT:PSS, to form an in‐series vertical structure. The bilayer design allows precise control over the threshold voltages for turning the device on and off, as well as the position of the peak current. This configuration not only reduces the overall device size but also enables the development of tunable threshold spiking neurons and logic gates. Additionally, the researchers demonstrated a retinal pathway, which successfully reproduced the encoding of wavelength and light intensity by horizontal cells, mimicking the behavior of spiking retinal ganglion cells.^[^ ^7^ ^]^

## 3 Printing Technologies for Additive Manufacturing of OECTs

Current OECT fabrication methods based on photolithography face challenges due to the incompatibility of conductive polymers with photoresist solvents and the complexity of multi‐step C‐parylene coatings. To address these limitations, recently Huang et al., demonstrate a micropatterning approach for organic semiconductors using electron beam exposure.^[^ ^21^ ^]^ This method converts exposed regions into electronic insulating layers, resulting in vOECT arrays with transconductance reaching 1.7 S and transient response times below 100 microseconds. However, electron beam exposure is a slow process, hampering large‐scale production. As an alternative approach, very recently Frusconi et al. proposed a scalable photolithographic process for large‐scale and simple microfabrication of OECTs.^[^ ^170^ ^]^ The method uses a two‐layer photoresist with controlled cross‐linking for precise polymer channel patterning and electrode encapsulation. The process yields reproducible and high‐performance OECTs, enabling their integration with polarizable gates and ion detection with sensitivity normalized to supply voltage of up to 6550 mV V^−1^ dec^−1^. Although photolithography allows precise and scalable micro‐patterning, it requires photoresists and solvents orthogonal to the OECT channel, and ad‐hoc material engineering is required. Moreover, photolithography requires significant amounts of solvents and materials, that are wasted after the development step. This has a negative impact on the environment and fabrication costs.

In contrast, additive manufacturing techniques eliminate the need for photomasks, reduce material waste, and are generally more cost‐effective for both small‐scale and large‐scale production. In addition, additive manufacturing enables seamless integration with flexible materials, broadening the scope of OECTs. In this section, additive manufacturing approaches used for OECT fabrication will be discussed, including inkjet printing, screen printing, and other advanced additive manufacturing methods. Specifically, the milestones of OECTs fabricated with additive manufacturing methods and their application fields and key achievements are analyzed and commented. The analysis highlights that additive manufacturing approaches can offer tailored device designs, optimized performance, and rapid prototyping capabilities.

### 3.1 Inkjet Printing

**Figure 5:**
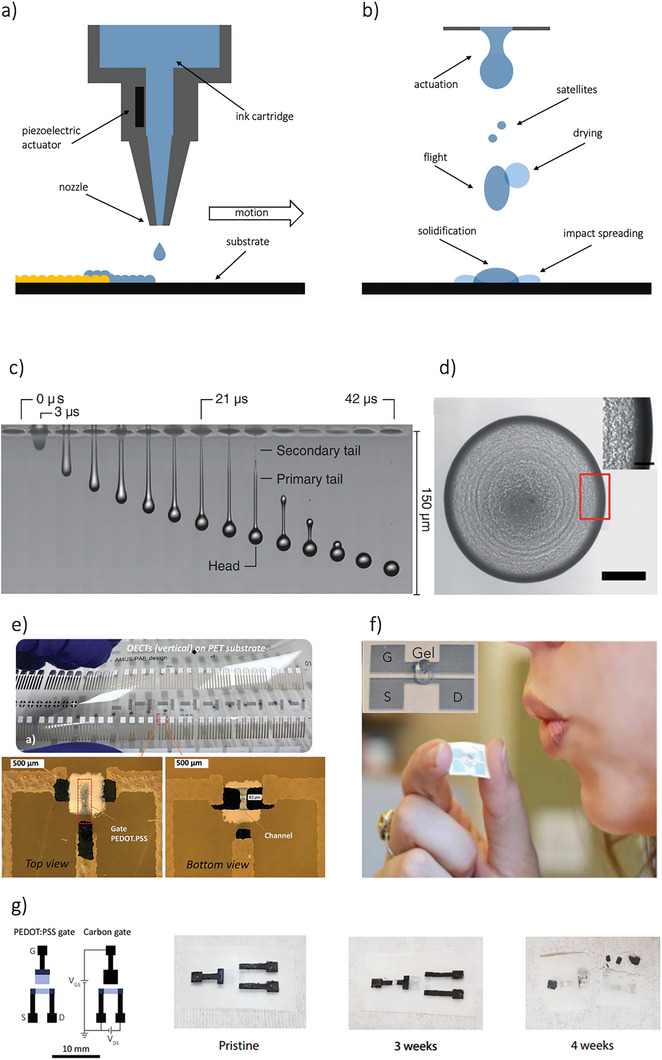
a) Schematic representation of the ink‐jet printing technique. The ink release through the nozzle is controlled by an actuator (typically a piezoelectric component). b) Schematic representation of the ink droplets evolution throughout the printing process, encompassing stages from actuation to impact with the substrate. Initially, during the flight phase, the ejected ink droplet moves through the air, detached from both the nozzle and the substrate. This phase highlights the presence of smaller satellite droplets and shows the onset of solvent drying (indicated in light blue) as the solvent begins to evaporate. Subsequently, the impact phase captures the interaction of the ink droplet with the substrate. This stage demonstrates ink spreading upon contact and the solidification process driven by continued solvent evaporation. c) Time series of droplets recorded with single‐flash photography. Adapted with permission.^[^ ^49^ ^]^ Copyright 2014, American Physical Society. d) Scanning electron microscopy (SEM) image of printed GO ink droplet on Si/SiO2 substrate after drying and showing a coffee ring. Scale bar is 100 µm. Adapted with permissions.^[^ ^171^ ^]^ Copyright 2017, Wiley‐VCH. e) All‐printed vertically stacked OECTs on top of a PET substrate. Transistors were fabricated by combining screen and inkjet printing. Adapted with permissions.^[^ ^66^ ^]^ Copyright 2024, Wiley‐VCH. f) Fully inkjet printed OECTs for disposable alcohol sensors on paper. Adapted with permissions.^[^ ^71^ ^]^ Copyright 2016, Springer Nature. g) Design of the degradable OECTs, and degradation of the transient device in soil at different time points. Adapted with permissions.^[^ ^69^ ^]^ Copyright 2023, Springer Nature.

Inkjet printing is one of the most prominent forms of non‐contact, on‐demand patterning. It requires low‐viscosity inks (typically in the range of 10 cP) and is commonly used to deposit metal nanoparticles and semiconducting polymer inks. The ink is ejected from the nozzles, flies through the air, impacts the substrate, spreads, and finally the solvent evaporates as the ink dries (**Figure** **5**a). The volume of ink droplets typically ranges from 1 to 100 picoliters, corresponding to a diameter of 10–100 µm. However, the low viscosity of the inks causes the droplets to spread after hitting the substrate, limiting the resolution of printed patterns (Figure 5b). While direct inkjet printing typically produces lines with a minimum width of several tens of micrometers, it is possible to reduce the space between patterns to less than 10 µm. This drop‐on‐demand, mask‐less technology allows for high‐throughput, large‐area patterning on customizable substrates. Inkjet printing has gained significant attention for its simplicity and versatility in patterning conjugated polymers. Most work on inkjet printing of polymers for OECTs has used piezoelectric print heads, where small drops of fluid are ejected through fine nozzles by pressure pulses from vibrating piezoelectric crystals. This method is widely recognized for its accuracy, repeatability, and simplicity. In addition to OECTs, inkjet printing has been used to create electronic components such as electrolyte‐gated transistors (EGTs),^[^ ^91^ ^]^ organic field‐effect transistors (OFETs),^[^ ^51^ ^]^ temperature sensors,^[^ ^52^ ^]^ and biosensors. Inkjet printing faces challenges related to droplet velocity, volume, and the presence of satellite droplets. The dynamics of picoliter‐sized droplets, from formation to impact, have been thoroughly studied through image processing (Figure 5c) and numerical simulations.^[^ ^49^ ^]^ A common issue that impacts the uniformity of printed patterns is the “coffee ring” effect (Figure 5d), inherently due to the drying of the solvent containing solutes or suspended particles.^[^ ^171^ ^]^

An important application of inkjet printing technology lies in its integration with standard photolithography to achieve faster response times in solid‐state OECTs. Meler et al. compared OECTs fabricated using three methods: pure photolithography, pure inkjet printing, and a hybrid approach that combines photolithographic patterning of the PEDOT:PSS channel with inkjet printing of the solid‐state electrolyte (SSE). The use of inkjet printing allows for better control of the SSE's height profile, resulting in improved OECT performance, particularly in terms of transconductance and reduced hysteresis.^[^ ^172^ ^]^ Inkjet printing is not limited to fabricating OECT channels and electrolytes; it is also used for printing electrodes. For example, recent experiments have implemented interdigitated planar side gated OECTs using this method.^[^ ^39^, ^173^ ^]^ This design achieved a channel ratio (W d / L) of 1258 µm and a maximum current of 1.23 mA, further enhancing OECT performance. Furthermore, Makhinia's group developed all‐printed accumulation mode organic electrochemical transistors via a combination of inkjet and screen printing technologies. The resulting OECTs (Figure 5e) demonstrated good switching performance, with high normalized transconductance (g_m, norm_ ≈13 mS cm^−1^), good mobility (µC* ≈ 21 F cm^−1^ V^−1^ s^−1^), and an ON–OFF ratio exceeding 10^4^, with stable cycling performance during continuous operation over 2 h. For the inkjet printing of pgBTTT (poly([4,4′‐bis(2‐(2‐(2‐methoxyethoxy)ethoxy)ethoxy)‐2,2′‐bithiophen‐5,5′‐diyl]‐alt‐[thieno[3,2‐b] thiophne 2,5‐diyl])), the polymer was first dissolved in dihydrolevoglucosenone (Cyrene), a non‐toxic, cellulose‐derived, biodegradable solvent. The resulting ink formulation exhibited excellent printability, enabling the production of reproducible and stable p‐type accumulation‐mode OECTs with high performance.^[^ ^66^ ^]^ The application of biodegradable and non‐toxic solvents, as an alternative to chloroform, is essential to decrease the environmental impact of technology also considering the unavoidable dispersion of electronic and bioelectronic devices in the environment. To this aim, Bihar and colleagues introduced an innovative disposable alcohol biosensor based on OECTs printed on a paper substrate (Figure 5f).^[^ ^71^ ^]^ Following a similar approach, fully inkjet‐printed glucose sensors on a paper substrate have been demonstrated.^[^ ^70^ ^]^ These devices demonstrate the potential for simple fabrication of robust and environment‐friendly sensors that can be integrated into portable devices, enabling various security and monitoring applications through IoT (Internet of Things) technology. However, these designs still relied on non‐degradable contacts for the printed OECTs' operation. To address this limitation, Khan et al. developed fully printed OECTs using a combination of blade casting and inkjet printing on cellulose acetate substrates with a processing temperature lower than 60 °C.^[^ ^99^ ^]^ Importantly, cellulose acetate is biodegradable and biocompatible, making it an ideal choice for green and transient electronics and bioelectronics. A noteworthy application of OECT fabrication, combining screen printing and inkjet printing, was presented by Fumeaux et al.^[^ ^69^ ^]^ They developed OECTs made from biodegradable materials, functioning as disposable biochemical sensors, utilizing carbon and PEDOT:PSS deposited on PLA substrate (Figure 5g). Further studies focused on optimizing the gate materials and areas, resulting in a maximum transconductance of 0.389 mS. Moreover, glucose detection was assessed, demonstrating the integration of the OECTs with highly conductive biodegradable zinc traces and interconnections. These eco‐friendly OECTs have the potential to serve as disposable and sustainable biochemical sensors, marking a significant advancement toward the development of bioresorbable biosensors.^[^ ^103^ ^]^

### 3.2 Aerosol Jet Printing and Spray Coating

**Figure 6:**
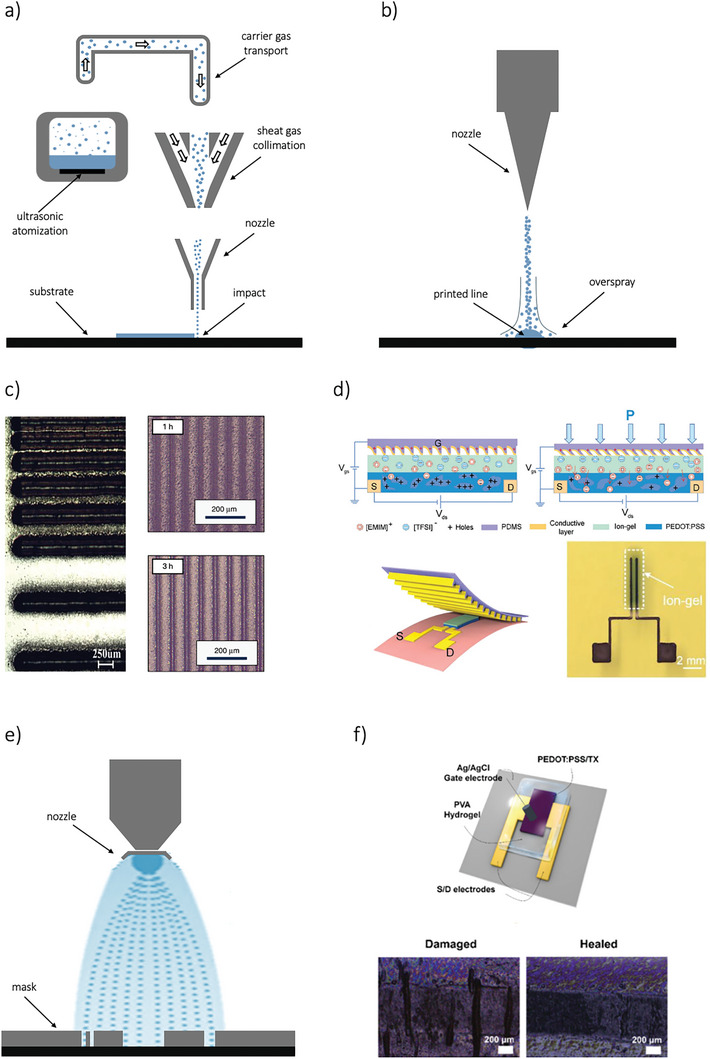
a) Schematic of the key physical processes involved in aerosol jet printing. b) Scheme of the overspray problem related to that technology. c) Overspray effect in an array of lines with various line widths. Adapted with permissions.^[^ ^128^ ^]^ Copyright 2018, Wiley‐VCH. Adapted with permissions.^[^ ^176^ ^]^ Copyright 2023, Springer Nature. d) A schematic representation of the tilting micro‐riblets printed via aerosol jet at the gate, illustrating how this tilting alters the gate‐electrolyte capacitance and thereby influences the OECT response. Adapted with permissions.^[^ ^182^ ^]^ Copyright 2023, Wiley‐VCH. e) Schematic representation of the spray coating technique. f) Self‐healable OECT fabricated through spray coating method. Adapted with permissions.^[^ ^62^ ^]^ Copyright 2020, American Chemical Society.

Aerosol jet printing has gained recognition as a highly promising method for microscale digital additive manufacturing, utilizing functional nanomaterial inks. This technique allows the deposition of inks with low and medium viscosity, typically in the range 1–1000 cP. The key operating principle involves balancing the pressure applied to the ink with the pressure driving its flow during its transit to the substrate. Aerosol jet printing can be broken down into three main stages: atomization of the ink, controlled flight and impact, and solvent drying. This fabrication method allows for easy variation in the width and thickness of printed traces, enabling either the rapid coverage of large substrate areas or precise, on‐demand maskless designs. The printed traces are usually in the range of tens of microns. As displayed in **Figure** **6**a, the aerosol jet printing process begins with ultrasonic or pneumatic atomization, where active ink droplets are generated. These micrometer‐sized droplets experience rapid solvent evaporation, which reduces their size. A carrier gas then transports the droplets to the deposition head. During this transit, droplet loss can occur through gravitational settling or diffusion onto the walls of the tube. Once at the deposition head, the aerosol gas is surrounded by another gas that helps to collimate the beam. As the droplets pass through a converging deposition nozzle, aerodynamic focusing occurs, potentially diverting droplets due to inertial effects. Finally, the droplets are directed toward the substrate in an impacting jet. To prevent complete drying of the aerosol droplets during the process, inks are typically designed with around 10% of a low‐volatility cosolvent, ensuring that the droplets retain enough solvent for proper deposition and pattern formation.^[^ ^174^ ^]^ The primary limitations of aerosol jet printing include ink spread and the overspray effect, which can lead to imprecise trace widths and issues with low‐gap traces (Figure 6b). These problems arise due to the size‐dependent trajectories of ink particles. Specifically, the effective line width is influenced by the mass accumulation of the ink, while overspray is mainly caused by particles originating from the outer edge of the aerosol jet stream (Figure 6c)^[^ ^128^ ^]^ and from the AJP parameters such as the focusing ratio,^[^ ^175^ ^]^ or the time of use.^[^ ^176^ ^]^ Another challenge involves potential nozzle fabrication and assembly defects or partial clogging in the flow passage. This occurs when ink particles adhere to the inner walls, disrupting the symmetry of the ink stream flow. Despite these challenges, aerosol jet printing has proven to be a versatile and effective method in printed electronics. For instance, it has been successfully employed to manufacture antennas from inorganic materials,^[^ ^177^ ^]^ to print components for organic field effect transistors,^[^ ^178^ ^]^ and to create passive electronic components.^[^ ^179^ ^]^ More recently, aerosol jet printing has also been used for printing biomaterials,^[^ ^180^ ^]^ as well as for producing stretchable and flexible electronics.^[^ ^181^ ^]^ In the context of OECT fabrication, Makhinia et al. highlighted the resolution advantage of aerosol jet printing (AJP) by producing fully printed OECTs through a combination of screen printing and AJP. They demonstrated that AJP could significantly reduce the OECT channel width (W) from 158 µm to just 15 µm, leading to notable improvements in the device's electrical characteristics, particularly in switching response times. Building on this architecture, they developed a fully printed five‐stage ring oscillator using the combined screen printing and AJP approach.^[^ ^54^ ^]^ In related work, Tarabella et al. advanced fully printed PEDOT:PSS‐based OECTs using the aerosol jet technique. Their focus was on minimizing overspray and improving the interface between the substrate and polymer, addressing key challenges associated with AJP.^[^ ^41^ ^]^

Subsequently, Zhou et al. utilized AJP to develop OECT‐based multi‐dimensional force sensors with tunable sensitivity and range, incorporating an unilaterally conductive micro‐riblet array. As shown in Figure 6d, the sensor features a micro‐fabricated gate formed by this riblet array. A key aspect of this design (top gated planar OECT) is the precise and repeatable fabrication of the AJP gate electrode, consisting of an array of gold micro‐riblets (300 µm in height and 500 µm in spacing). When mechanical force, such as pressure, is applied, the riblets tilt, forming an unilaterally conductive layer that alters the OECT's electrical response based on capacitance changes at the gate‐electrolyte interface. The further integration of four OECTs enables the sensor to detect the direction of the applied force, which has potential applications in intelligent robotics and human‐machine interaction.^[^ ^182^ ^]^ AM AJP's non‐contact printing capability offers significant advantages for OECT fabrication by simplifying and streamlining the process. This approach reduces complexity and facilitates the seamless integration of diverse materials. These benefits are especially valuable for applications such as integrating complementary OECTs into flexible neural implants,^[^ ^25^ ^]^ or developing tunable vertical anti‐ambipolar OECTs for neuromorphic systems.^[^ ^7^ ^]^ Current fabrication methods for these applications involve intricate, multi‐step processes, including the direct deposition and peel‐off of parylene layers on the active channel. By contrast, AJP technology can simplify these workflows, reduce fabrication challenges, and enhance device performance.

Similarly to aerosol jet, spray coating is a non‐contact technique allowing material deposition based on atomization. The material passes from the liquid state to a gaseous state and a cone flow of particles is generated. The most significant advantage of spray coating is its compatibility with large‐area industrial scale processes although the deposit of micrometric‐sized structures requires masks (Figure 6e). Recently, spray coating has been employed to fabricate complementary OECTs, particularly for printing the channel layers. For instance, Fabiano et al. used PEDOT:PSS ink and an n‐type polymeric ink, a blend of BLL and PEI, to develop complementary logic OECTs, both in depletion mode. These devices were directly printed onto shadow‐masked gold electrodes on a glass substrate. The same technology was applied to demonstrate the usability of these inks in thermoelectric generators (TEGs), achieving a record‐high power output of 56 nW per p–n pair at a temperature difference (ΔT) of 50 K.^[^ ^63^ ^]^ Building on this, the same research group demonstrated fully printed OECT‐based complementary circuits. They combined screen printing to create source/drain carbon‐silver electrodes and PQ‐10 (polyquaternium‐10) and PSS:Na hydrogel electrolytes, while using spray coating to deposit p‐type enhancement‐mode P(g42T‐T) and n‐type enhancement‐mode BBL inks, along with a side Ag/AgCl gate electrode. The resulting p‐ and n‐type OECTs achieved maximum transconductances of 0.17 mS and 0.18 mS, respectively. These circuits included flexible integrated complementary inverters with a gain of 26 V/V, maximum power consumption of 10–15 µW, and static power consumption of 1.35 µW at VDD = 0.6 V, as well as the produced OECT‐based two‐stage push‐pull amplifier with a high gain of 193 V/V.^[^ ^22^ ^]^ A noteworthy application of spray coating involves the integration of a non‐polarizable Ag/AgCl gate into OECTs fabricated through pure photolithography. Recently, Lim et al. developed monolithic tandem vertical OECTs for multi‐valued logic operations, produced by stacking a bilayer of PEDOT:PSS and P3HT with a spray‐coated side Ag/AgCl gate.^[^ ^26^ ^]^ Similarly, Moon et al. fabricated spray‐coated top‐gate vertical PEDOT:PSS‐based OECTs, with a maximum frequency of 12 MHz, a maximum transconductance of 30 mS, and a channel length of 50 nm.^[^ ^24^ ^]^

An interesting application of spray‐coated OECT channels consists of printing self‐healable materials. For example, Ko and Wu group provide the first report of an all‐solid‐state OECT that is self‐healable and possesses good electrical performance, by using a matrix of PEDOT:PSS and a nonionic surfactant, Triton X‐100, as a spray coated channel and an ion‐conducting poly(vinyl alcohol) PVA hydrogel as a quasi‐solid‐state polymer electrolyte (Figure 6f). The fabricated OECT exhibits high transconductance (54 mS), an on/off current ratio of ≈1.5 × 10^3^, a fast response time of 6.8 ms, and good operational stability after 68 days of storage. In addition, due to the swelling of the polymer and the amphiphilic surfactant, the spray coated OECTs showed remarkable self‐healing (> 95%) as well as good ionic transport and electrical performance after mechanical damage.^[^ ^62^ ^]^ The development of self‐healing electronic materials, which can repair themselves like living tissue, is vital for the future of electronics. These materials offer the critical ability to self‐repair and maintain durability, especially in wearable devices, where stretching and cracks caused by wear and tear are common. In this direction, Su et al. spray‐coated a polymer composed of PEDOT:PSS mixed with poly(2‐acrylamido‐2‐methyl‐1‐propanesulfonic acid), PAAMPSA and an ionic liquid (IL) of 1‐Ethyl‐3‐methylimidazolium – trifluoromethanesulfonate (EMIM OTF), to create tactile OECT sensors channel with silver nanowires (AgNWs) source/drain electrodes and single‐walled carbon nanotube (SWCNT) gates. The fabricated OECTs showed transconductance of 13 mS, fast transient response of 7.1 ms, good on/off ratio of ≈ 10^3,^ and self‐healable characteristics with a polymer maximum strain of 600%.^[^ ^183^ ^]^

### 3.3 Screen Printing

**Figure 7:**
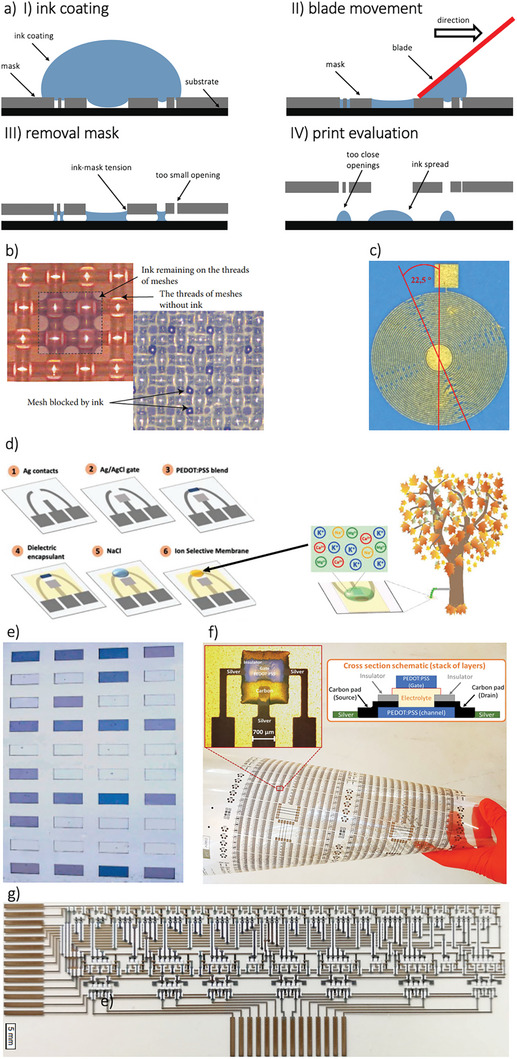
a) Schematic representation of the screen printing fabrication method. I) Ink coating on all the mesh mask. II) Forcing ink through the mesh with a squeegee movement and removal of the exceeding ink. III) Careful removal of mask from the substrate. The tension between ink and mask depends mostly on its viscosity. IV) Resulting print and their problems, such as the union of two or more patterns because openings are too close in the mask pattern and the presence of too small openings that avoid the ink deposition. b) Example of traces printed with screen printing technique. Smaller apertures will cause the conductive ink to block the screen. Adapted with permissions.^[^ ^194^ ^]^ Copyright 2022, Wiley‐VCH. c) Screen printed failure (Moire‐effect) in structures like circles or coils (500 mesh/18 µm, cal. Angle 22.5°). Adapted with permissions.^[^ ^186^ ^]^ Copyright 2009, International Microelectronics and Packaging Society. d) Schematic structure and order of the screen printed layers for ion‐sensitive OECT in plant sap electrolyte. Adapted with permissions.^[^ ^133^ ^]^ Copyright 2021, Wiley‐VCH. e) 4 × 10 active matrix paper display with smart pixel cells realized on polyethylene‐coated paper. Adapted with permissions.^[^ ^78^ ^]^ Copyright 2002, Wiley‐VCH. f) Screen‐printed sheet containing 760 OECTs, a microscope image of a single OECT, and a cross‐section schematic illustrating the different material layers of the OECT architecture. Adapted with permissions.^[^ ^37^ ^]^ Copyright 2020, Springer Nature. g) All‐printed decoder with 87 PEDOT:PSS‐based OECTs. Adapted with permissions.^[^ ^20^ ^]^ Copyright 2019, Springer Nature.

Screen printing is one of the most widely used techniques for printing electronics, relying on a process known as “stenciling,” in which ink is pushed through a screen using a blade or squeegee (**Figure** **7**a). The ink adheres to the substrate, forming a printed pattern. This method is highly efficient and allows for fast production, with easy alignment of multiple layers due to the use of stencils. Typically, the minimum printed trace widths are in the range of tens of microns, and the layer thickness is usually a few microns. However, low‐viscosity inks can lead to undesirable material flow through the mesh, reducing pattern resolution. As a result, screen printing is better suited for high‐viscosity inks/pastes, typically over 10000 cPs.^[^ ^184^ ^]^ Paste thickness of the printed material is determined by the size and number of openings in the mesh; finer meshes produce thinner prints, while coarser meshes lead to thicker layers. Additionally, the speed and angle of the squeegee influence the amount of ink transferred to the substrate. Faster squeegee speeds create more pressure on the mesh, releasing larger amounts of ink,^[^ ^185^ ^]^ but excessive speed can lead to incomplete ink transfer, resulting in blocked mesh and faulty layers (Figure 7b,c). Smaller squeegee angles also increase ink deposition. To solve the problem of ink transferring rate reduction, several adjustments can be made, such as modifying the screen structure,^[^ ^186^ ^]^ altering the rheological properties of the conductive ink,^[^ ^187^ ^]^ or refining the printing parameters.^[^ ^188^ ^]^

Screen printing technology requires a significant amount of ink during the fabrication process, but its speed, simplicity, and ability to handle large‐area substrates make it the primary method for fabricating fully printed OECTs. This technique is often employed in producing logic gates, complex circuits, and high‐speed OECT‐based systems.^[^ ^95^, ^97^, ^189^ ^]^ In these studies, both bottom‐ and top‐contact planar OECTs were fabricated, where carbon paste source/drain electrodes were partially printed onto the PEDOT:PSS channel. The electrical characteristics of the OECTs were analyzed by varying the covered area of the channel, focusing on factors such as fast switching time and off‐current behavior. To further showcase the potential of screen‐printed OECTs, Scheiblin et al. developed a referenceless pH sensor. This sensor utilized OECTs fabricated by screen printing the active PEDOT:PSS channel and a dielectric layer to protect the gold interconnection layer. Specifically, they created an OECT‐based Wheatstone bridge for differential pH measurements, with the key innovation being the use of a reference solution rather than a traditional reference electrode.^[^ ^190^ ^]^ In more recent work, Galliani et al. developed an OECT biosensor on PET foil for real‐time healthcare monitoring, specifically to detect uric acid, a biomarker for bacterial infections in wounds. The sensor, demonstrated in artificial wound exudate, achieved a limit of detection (LoD) of 4.5 µM.^[^ ^190^ ^]^ Most representative sensors produced via screen printing were developed by Strand et al., introducing a fully screen‐printed OECT designed for detecting nutrients in whole plant sap. This work features an ion‐selective OECT capable of measuring macronutrient concentrations in plant sap, with potassium selected as the target analyte due to its critical role in plant growth and development. The ion sensors exhibit impressive performance, high current (170 µA dec^−1^), and voltage (99 mV dec^−1^) sensitivity, and a low limit of detection (10  ×  10^−6^ M). Particularly, a polyvinyl chloride (PVC)‐based ion‐selective membrane (ISM) was incorporated between the electrolyte and the PEDOT:PSS channel to sense targeted ions in the aqueous analyte (Figure 7d).^[^ ^133^ ^]^ Interestingly, Makhinia and colleagues embedded a screen‐printed OECT with inkjet‐printed hydrophilic coatings to facilitate flow control in microfluidic applications.^[^ ^191^ ^]^ This innovative approach involves applying a carefully designed nonhomogeneous coating that creates regions with varying wetting properties on the microchannel walls. This variation alters the curvature of the liquid‐air meniscus at different channel cross‐sections, leading to different capillary pressures and enabling automatic flow control. By implementing this method, the researchers developed “stop” and “delay” valves, which allow for preprogrammed capillary flow and the sequential release of fluids. The system demonstrated sensing capabilities through the integration of screen‐printed OECTs within the microfluidic chips, enabling the sequential and independent detection of chloride anions with a concentration in the range 1–100 × 10^−3^ M.

The most significant applications of screen‐printed OECTs are in logic circuits and large‐area production. Over the past decade, the size and complexity of printed circuits have steadily increased, expanding the potential of OECT‐based circuits into new fields. For example, Andersson et al. proposed all‐printed electrochromic displays (ECDs) utilizing a pixel active matrix based on PEDOT:PSS electrochemical transistors (Figure 7e),^[^ ^78^ ^]^ also assuming the possibility of manufacturing active matrix displays in a roll‐to‐roll production procedure.^[^ ^79^ ^]^ Following a similar approach, Boda et al. developed an electrochromic smart pixel by integrating fully screen‐printed stretchable OECTs with a stretchable electrochromic display. The stretchable OECTs demonstrated excellent performance, maintaining reliable transfer curves, output characteristics, and transient responses under up to 100% static strain and enduring 500 strain cycles at 25% and 50% strain.^[^ ^38^ ^]^ Zabihipour and co‐workers addressed the high‐yield potential of screen printing for OECT fabrication, successfully producing a large‐scale integration of 760 OECTs with varied characteristics on a PET sheet (Figure 7f).^[^ ^37^ ^]^ This demonstrates the scalability of the screen‐printing technique for OECT manufacturing. Moreover, several experimental studies have successfully demonstrated the fabrication of OECT‐based logic gates and building blocks for digital electronics, including components such as NOT gates, NAND gates, organic ECD, and ring oscillators through mixing screen, inkjet, and aerosol jet printing.^[^ ^54^, ^95^, ^97^, ^192^ ^]^ Additionally, recent advancements have showcased large‐area, screen‐printed logic circuits, including a seven‐bit shift register composed of 114 OECTs and a binary‐coded decimal decoder featuring 87 OECTs. Gustafsson et al. reported the development of screen‐printed vertical OECTs with channel dimensions of W × L × d = 200 × 200 × (0.3–0.4) µm^3^, where the electrolyte vertically bridges the bottom channel and the top gate electrode (Figure 7g).^[^ ^20^ ^]^ These OECTs operate at low voltages (1–3 V) and deliver high current throughput (in the mA range), making them effective for controlling light emission in LEDs, in which the actual LED addressing was accomplished by an OECT‐based decoder circuit.

Recently, Zabihipour et al. addressed the low‐resolution issues associated with the screen printing technique by combining it with laser ablation to produce OECTs with a minimum channel length of 25 µm.^[^ ^98^ ^]^ A similar technique was also previously demonstrated by Rashid et al. in their work on ambipolar inverters, which relied on cofacial pairs of vertical organic electrochemical transistors for biosignal amplification.^[^ ^193^ ^]^ Notably, in Rashid's work, laser ablation was specifically employed to create structural cuts essential for developing vertical OECTs fabricated via photolithography, showcasing its potential in advancing device architecture. Using this approach, traditional photolithographic processes could be potentially replaced by combining screen printing with laser ablation, facilitating the development of ultra‐low‐cost and high‐performance OECTs.

### 3.4 Transfer Printing, Gravure Printing, and Flexography

**Figure 8:**
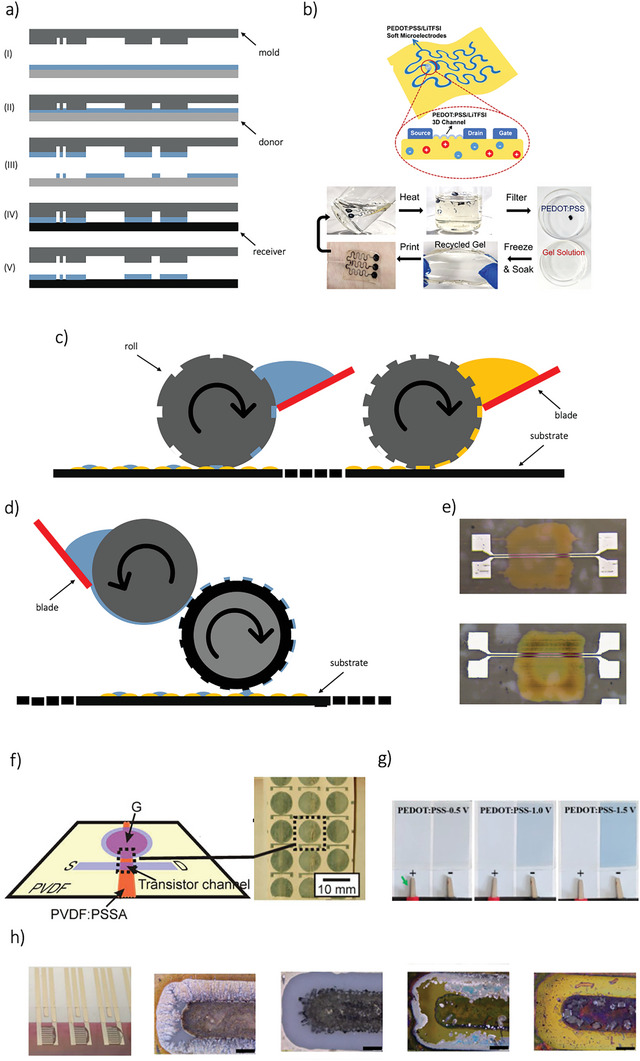
a) Schematic of transfer printing. b) Substrate‐free all‐polymer OECT utilizing gelatin‐glycerol/sodium citrate (GEL‐GLY/Na3Cit) organohydrogel electrolyte as the elastic substrate, and PEDOT:PSS/LiTFSI as both the soft electrodes and the active channel layer with 3D micro‐engineered interface. Recycling of the all‐polymer OECT and performance of the OECT using the recycled gel electrolyte. Adapted with permissions.^[^ ^201^ ^]^ Copyright 2022, Springer Nature. c) Schematic of the gravure printing. d) Schematic of the flexography printing. e) Example of semiconductor pattern spreads by varying the ink viscosity (top is 36 cPs, bottom is 130 cPs) in a gravure printing process. Adapted with permissions.^[^ ^204^ ^]^ Copyright 2016, Wiley‐VCH. f) Flexography printed al polymer sensors on PVDF:PSSA membrane. Adapted with permissions.^[^ ^206^ ^]^ Copyright 2010, Elsevier. g) Photographs of a PEDOT:PSS electrochemical display (ECD) with pixel color varying with the applied voltage. Adapted with permissions.^[^ ^215^ ^]^ Copyright 2022, Elsevier. h) R2R printed OECT after testing, from left to right, representative image demonstrating silver electrode etching after testing. Optical micrograph of the bare electrode structure after testing in DI water. Optical micrograph of Nafion/ P3HT/Ag structure after testing in DI water. Optical micrograph of Nafion/P3HT/ Ag structure after testing in 10 mM PBS solution. Scale bars are 100 µm. Adapted with permissions.^[^ ^218^ ^]^ Copyright 2024, AIP Publishing.

Transfer printing is a contact printing technique that involves transferring material from a “donor” substrate to a “receiver” substrate using a mold (**Figure** **8**a). This method provides an effective alternative to traditional direct film handling and molding techniques. Consequently, significant efforts have been made to transfer print OMIECs onto soft substrates.^[^ ^195^, ^196^, ^197^ ^]^ For instance, Greco et al. developed a technique where PEDOT:PSS films were initially deposited on PDMS.^[^ ^196^, ^198^ ^]^ They successfully achieved the transfer of ultrathin PEDOT:PSS films onto soft substrates by taking advantage of the gradual degradation of adhesion forces between the PEDOT:PSS and PDMS over time. Similarly, Bihar et al. demonstrated the application of PEDOT:PSS on the skin by transferring inkjet‐printed PEDOT:PSS films to tattoo paper.^[^ ^199^ ^]^

In the context of OECT fabrication, Zhang et al. presented a hydrogel‐enabled transfer printing, which facilitated the creation of conducting films for soft skin‐attachable OECTs. These OECTs were specifically designed for glucose detection, showcasing the versatility and potential of transfer printing in developing advanced electronic applications. In this process, a carrier gel such as PVA^[^ ^198^ ^]^ or PDMS^[^ ^200^ ^]^ is used to lift the PEDOT:PSS pattern from a donor substrate, such as glass,^[^ ^196^ ^]^ to a target substrate. Using the same method, PEDOT:PSS pads were transferred on tattoo paper populated with sputtered gold electrodes, and then attached directly to the skin.^[^ ^139^ ^]^ Very recently, Wang et al. reported the development of elastic all‐polymer OECTs featuring high transconductance levels of up to 12.7 mS, along with long‐term mechanical and environmental durability. These OECTs were created by transfer printing lithium bis(trifuoromethane) sulfonimide doped PEDOT:PSS (PEDOT:PSS/LiTFSI) microstructures onto a resilient gelatin‐based gel electrolyte. In this configuration both polymer electrodes and polymer channel were deposited on PDMS mold and transferred onto an electrolyte‐gel substrate that can be directly attached to the human skin, with these OECT they demonstrated the operation under a wrist bending to control a LED brightness and the Long‐term potentiation (LTP) and Long‐term depression (LTD) synaptic behaviors. Notably, the resulting all‐polymer OECTs and gelatin‐based organohydrogel electrolyte substrates were fully recyclable, through immersion in hot water and filtration (Figure 8b).^[^ ^201^ ^]^

As an evolution of transfer printing, gravure printing, and flexography are among the fastest printing technologies, achieving speeds between 100–1000 m min^−1^.^[^ ^202^ ^]^ The gravure printing process can be divided into three main steps: first, the cells engraved on the roll are filled with ink; second, any excess ink is removed from the surface; and third, the ink remaining in the engraved cells is transferred to the substrate (Figure 8c). Flexography printing employs an additional printing plate cylinder, usually made from polymer or plastic, which contains a mold of the desired characteristics (Figure 8d). Both gravure and flexography printing techniques have their precision limited by the quality of the engraved or molded rolls, with typical precision around tens of microns. The primary advantage of gravure and flexography printing techniques lies in their ability to facilitate the mass fabrication of multilayer devices, albeit with some limitations in precision. To optimize the printing process, it is crucial to meticulously design and pattern the features on the roll to prevent ink residue from accumulating. Additionally, the blade placement must be carefully calibrated to avoid issues such as meniscus formation and ink spillage. The wettability of the substrate also plays a significant role in influencing ink diffusion, impacting the overall quality of the printed features (Figure 8e).^[^ ^203^, ^204^, ^205^ ^]^ The potential for large‐scale production of low‐cost electronic devices has sparked considerable interest in printed organic electronics. However, transitioning from laboratory‐scale production to industrial‐scale manufacturing poses significant challenges. This transition necessitates adapting materials and fabrication processes that are typically optimized for smaller scales to industrial techniques, such as roll‐to‐roll (R2R) and flexography printing. While the literature on using these technologies for printing electrochemical devices is limited, some notable works have emerged. For example, Kaihovirta et al. successfully demonstrated all‐polymer OECTs with worm memory functionality demonstrated through the PEDOT overoxidation, fully produced through flexography, particularly the transistors are directly printed on an ion conducting membrane of sulfonated poly(vinylidene difluoride) (PVDF:PSSA) (Figure 8f).^[^ ^206^ ^]^ Particularly, these printing methods, while widely utilized in various applications, are generally not very popular for producing active areas or electrodes for OECTs. Specifically, gravure and flexographic printing are well‐suited for large‐scale production runs that involve repeating a limited number of footprints multiple times. In the literature, they have demonstrated the use of adaptive roll‐to‐roll techniques for the production of organic solar cells,^[^ ^207^, ^208^ ^]^ and sensor components such as conductive electrodes,^[^ ^209^ ^]^ active polymers,^[^ ^208^, ^210^ ^]^ OMIECs,^[^ ^207^ ^]^ and solid‐state electrolytes.^[^ ^211^ ^]^ However, these methods are still limitedly used in the production of OECTs. Additionally, in the domain of electrochromic applications, these printing techniques play a significant role in the creation of smart labels and devices (Figure 8g).^[^ ^212^, ^213^, ^214^, ^215^ ^]^ They are also utilized in biosensing technologies for detecting amines and ions, specifically through the fabrication of the canonical working, reference, and counter electrodes essential for such sensors.^[^ ^216^ ^]^ A promising area in printed OECTs lies in memory components for neuromorphic applications. For Example, through the use of R2R printing, Grant's group developed a non‐volatile conjugated polymer‐based electrochemical memristor (cPECM) for boolean and elementary algebra operations, utilizing water‐soluble self‐doped PEDOT (S‐PEDOT).^[^ ^217^ ^]^ Specifically, the created 3‐terminal device developed used a “readout” channel in which the conductivity of the water‐soluble, self‐doped S‐PEDOT was equated with synaptic weight and was electrically decoupled from the programming electrode. For the model system, a +2500 mV programming pulse of 100 ms duration produced a resolution of 0.136 µS in conductivity change, providing more than 1000 distinct conductivity states for one cycle. They validated mathematical operations of addition, subtraction, multiplication, and division with a single cPECM, as well as AND, OR, NAND, and NOR logic gates. This demonstration of a printed cPECM is the first step toward the implementation of a mass‐produced electrochemical memristor that combines information storage and processing and can enable printable artificial neural networks. Additionally, Elkington employed roll‐to‐roll printing to fabricate both OECTs and glucose sensors (Figure 8h), further illustrating the versatility and potential of these techniques in electronic applications.^[^ ^218^ ^]^

Another breakthrough in printed electronics came from Mahajan et al., who demonstrated a self‐aligned strategy exploiting capillary flow on microstructured plastic surfaces. Using a microimprinted mold and inkjet printing, they fabricated micro‐sized channel OECTs by filling reservoirs for each material layer. This method addresses the resolution limitations of large‐area, high‐speed printing techniques like R2R, as it focuses on the precision filling of specific reservoirs, enabling the fabrication of high‐performance OECTs at scale.^[^ ^219^ ^]^

### 3.5 Extrusion Printing

**Figure 9:**
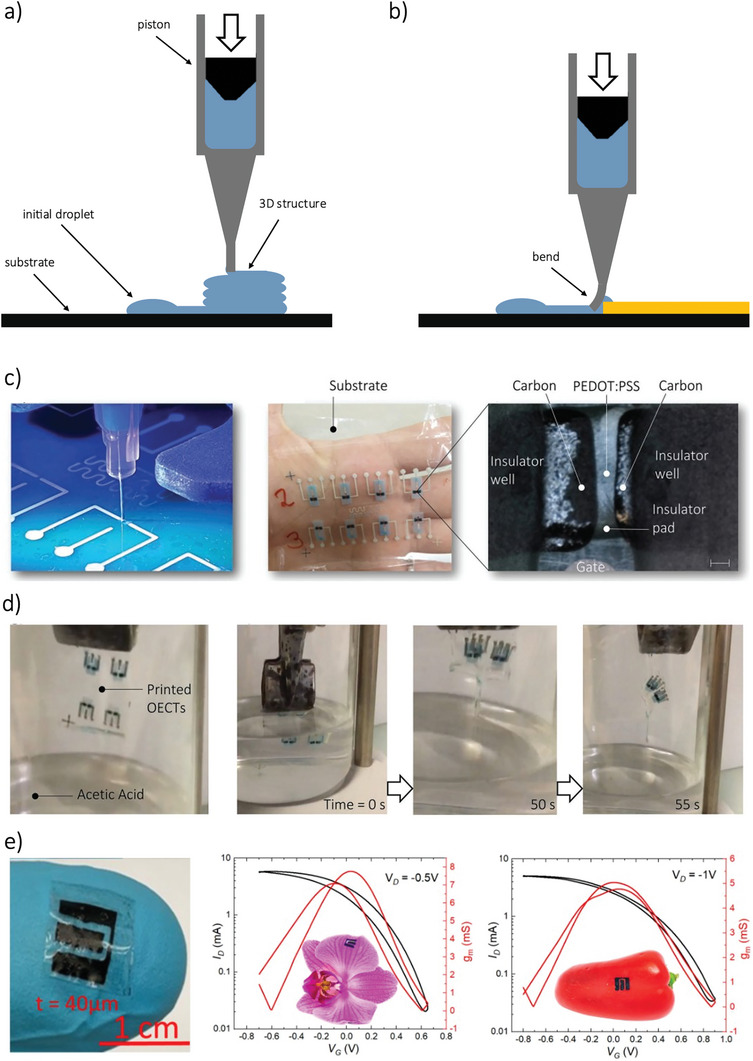
a) Schematic representation of extrusion printing technology. b) Typical issue when dispensing materials with previously deposited structures. c) From left to right, the photograph of the dispensing head operated by the gantry system. Various OECTs were printed on a diacetate cellulose substrate. Zoom at the center of the printed OECT channel. Scale bar is 200 µm. d) Fast degradation through immersion of the printed OECTs in acetic acid at a concentration equal to 90%, i.e., an environment‐friendly solvent. Dissolution after 50 and 55 s. Adapted with permissions.^[^ ^44^ ^]^ Copyright 2022, Wiley‐VCH. e) Photograph of the fully 3D printed OECT on a plastic substrate and directly printed on an orchid flower and bell pepper, including transfer curves and transconductance. Adapted with permissions.^[^ ^43^ ^]^ Copyright 2023, Springer Nature.

Extrusion printing technique, also referred to as dispensing or 3D printing for layered applications, and often known as capillary printing for single‐layer or 2D applications, is gaining significant attention across various fields, from bioprinting^[^ ^220^, ^221^, ^222^ ^]^ to the creation of soft and solid 3D structures using resins, metals, and ceramics.^[^ ^223^, ^224^, ^225^ ^]^ The fundamental principle of dispensing is material extrusion which involves controlling the flow of material from a source (e.g., a nozzle or syringe) onto a build platform in a way that forms the desired structure (**Figure** **9**a). The material is then bonded layer by layer to itself or to a secondary building material, ultimately forming a coherent solid structure. Additive manufacturing via material extrusion can be further classified based on the type of extruder used, including filament extrusion,^[^ ^226^, ^227^ ^]^ piston extrusion,^[^ ^221^, ^228^ ^]^ pneumatic extrusion,^[^ ^221^, ^229^ ^]^ and screw extrusion.^[^ ^223^, ^228^ ^]^ One of the advantages of extrusion‐based printing is its simplicity and affordability. This technique allows for a continuous flow of functional materials to be extruded through a nozzle, enabling the dispensing of high‐viscosity materials, typically greater than 10000 cP, while encountering fewer clogging issues compared to other printing methods. However, this approach is relatively slow and often suffers from lower printing resolution, which is largely determined by the nozzle dimensions and the characteristics of the ink, such as particle size and drying time. A significant benefit of dispensing is the material efficiency as it uses only the necessary amount of ink to achieve the desired pattern, thereby reducing waste. Typically, the inks used in dispensing can create traces with minimum widths on the order of tens of microns. Recently, this technique has been employed to fabricate fully printed OECTs on unconventional^[^ ^43^ ^]^ and biodegradable substrates,^[^ ^44^ ^]^ as well as in both planar and vertical structures.^[^ ^68^, ^93^ ^]^ Despite its advantages, material extrusion‐based printing does present challenges, such as ensuring accurate initial deposition during the first contact with the substrate, which can compromise print accuracy. Additionally, there are risks of bending or breaking the nozzle during the printing process (Figure 9b).

Dispensing technology has become a powerful tool for fabricating highly integrated 3D multifunctional constructs, driving extensive research into its applications in advanced structures and materials. A notable example comes from Yuk et al., who developed a 3D‐printable ink based on PEDOT:PSS through a combination of freeze‐drying and redispersion techniques. This process enabled the creation of intricate mesh structures, achieving a minimum needle size of 30 µm. The key to this method lies in optimizing the concentration of PEDOT:PSS nanofibrils, specifically within an intermediate range (5–7 wt.%), which ensures ideal rheological properties for 3D printability. The resulting hydrogel exhibited excellent electronic and mechanical properties, with a conductivity of 28 S cm⁻¹ and a Young's modulus of 1.1 MPa. Furthermore, they successfully printed a soft neural probe with nine channels, which was implanted in a mouse for in vivo electrophysiological recordings.^[^ ^60^ ^]^ Similarly, Zheng et al. introduced an innovative method to improve 3D printing of PEDOT:PSS by using a coagulation bath to enhance material performance. In this approach, the printing tip was submerged in a dodecyl benzene sulfonic acid (DBSA) bath during the printing process.^[^ ^61^ ^]^ leveraging the gelation effect and timing created by the DBSA's presence in the ink. Once the ink was extruded into the coagulation bath, the electrostatic attraction between the aligned PEDOT⁺ and PSS⁻ chains weakened due to the bath's high ionic strength. This allowed the exposed PEDOT⁺ chains to interact with DBSA micelles, forming an interconnected PEDOT:PSS network. To demonstrate the bioelectronics potential of this method, they printed a micro‐electrocorticography (µECoG) neural interface, which covers the cortex for intracranial electrical stimulation and simultaneous calcium imaging of brain activity in mice. The µECoG electrodes, positioned epidurally on the brain's surface, provided a high signal‐to‐noise ratio (SNR) and localized cortical signals, avoiding common complications of traditional intracortical neural probes, such as infection, biological rejection, and signal instability.

In the field of organic electrochemical transistors (OECTs), early research into fully 3D‐printed OECTs began a few years ago. Majak et al. pioneered the fabrication of a fully 3D‐printed inverter logic gate sensor using PEDOT:PSS as the channel material to function as an ion‐level detector. The device was tested with various electrolytes such as Sodium Chloride (NaCl), Potassium Chloride (KCl), and Calcium Chloride(CaCl₂), demonstrating its reliability and repeatability as a high‐performance cation sensor. It exhibited a sensitivity of 650 mV/dec in a concentration range of 1–100 mM for Na⁺ ions.^[^ ^230^ ^]^ Fan et al. also contributed by developing fully 3D‐printed OECTs on PLA, PDMS, and Polyethylene terephthalate (PET) substrates, achieving a maximum transconductance of 31.8 mS. These devices were used for pH and K⁺ ion sensing, with high sensitivity values of −26.4 mV/pH for pH sensing and 240 mV/dec for K⁺ ion detection.^[^ ^231^ ^]^ Both studies utilized commercially available PEDOT:PSS paste (viscosity > 50000 cP) along with bottom‐contact silver source and drain electrodes. Another noteworthy advancement involved the development of high‐performance bioelectronic circuits integrated into biodegradable and compostable substrates. These OECT circuits were fabricated by combining dispensing and capillary printing techniques (Figure 9c,d).^[^ ^44^ ^]^ A key innovation was the use of a carbon paste interlayer between the silver electrodes and the PEDOT:PSS liquid ink channel. This interlayer reduced redox reactions at the interface, resulting in improved device stability and lower off‐current. The fabricated inverters on diacetate cellulose film (degradation time less than three months in marine water and less than two months in industrial compost), demonstrated remarkable performance, with a gain normalized to the supply voltage reaching up to 136.6 V^−1^. This impressive performance was attributed to the bottom‐contact, side‐gated architecture of the printed OECTs, which allowed for precise control of channel dimensions. This enabled the design of inverters with optimal active area ratios between the drive and load OECTs. Additionally, the work highlighted the potential of these OECTs as ion sensors, achieving a high sensitivity of up to 506 mV/dec, further showcasing their versatility for bioelectronic applications. Furthermore, Massetti et al. introduced fully 3D‐printed OECTs with dimensions of 240 µm in width and 77 µm in length, achieving an impressive on/off ratio greater than 1000 and a transconductance of 8 mS. A key feature of this work was the ability to print these devices directly onto unconventional substrates, such as bell peppers and orchid flowers (Figure 9e). These OECTs demonstrated sensitivity to dopamine, thanks to the functionalized GO/CNT (a blend of graphene oxide (GO) and carbon nanotubes (CNTs)) gate ink, achieving a limit of detection (LoD) of 6 µM. Additionally, the devices exhibited neuromorphic behavior, with a long‐term synaptic response lasting approximately one hour, showcasing their potential for bioelectronic and neuromorphic applications.^[^ ^43^ ^]^ They were fabricated using both sides of a plastic or paper substrate. Carbon paste was used to create the top and bottom electrodes (source and drain, respectively) on either side of the substrate. This was accomplished by combining drop casting and screen printing on PET, as well as inkjet printing on paper. These devices featured a top‐gated, vertical edge‐channel structure, achieving on/off ratios of 4000 for PET substrates and 280 for paper one.^[^ ^232^ ^]^ A more recent advancement in fully 3D‐printed OECTs was presented by Azimi et al., who introduced the first fully printed vertical OECTs (V‐OECTs).^[^ ^68^ ^]^ In a comparison between planar and vertical architectures, the vertical OECTs demonstrated faster response times and higher transconductance due to their shorter channel length (≈2 µm). Electrochemical impedance spectroscopy (EIS) characterization revealed further insights, where two types of electrolytes were developed: an iongel made from poly(vinylidene fluoride)‐co‐hexafluoropropylene (PVDF‐HFP) and the ionic liquid ([EMIM][TFSI]), and an organogel based on hydrophilic PVA dissolved in an aqueous NaCl solution. The V‐OECTs showed impressive transconductance values—50 mS for the iongel and 130 mS for the organogel. Additionally, the vertical architecture achieved a more than fourfold reduction in the off‐time constant compared to planar devices, decreasing from 2.31 seconds to 0.55 seconds when using the organogel electrolyte. Moreover, recently Li et al. developed a home‐built printing system to fabricate intrinsically stretchable synaptic OECTs array by using extrusion printing technique.^[^ ^233^ ^]^ The resulting OECTs demonstrated high transconductance (22.5 mS), excellent mechanical softness (Young's modulus ≈2.2 MPa), and stretchability (≈30%). Notably, the devices also exhibited artificial synapse functionality, mimicking the biological synapse with features such as paired‐pulse depression, short‐term plasticity, and long‐term plasticity.^[^ ^202^ ^]^

A significant breakthrough was achieved by integrating the dispensing method with standard photolithographic processes to incorporate a non‐polarizable Ag/AgCl gate electrode. This advancement, demonstrated by Frusconi et al., was featured in recent work on selective, real‐time monitoring of floating‐gate OECTs. These devices were developed by combining a separated electrolyte well with an ion‐selective gate, allowing for precise ion sensing. Notably, the fabricated OECTs exhibited exceptional selectivity for K⁺ ions, with a detection limit as low as 11 × 10⁻⁶ M, even in the presence of Na⁺ ions at concentrations two orders of magnitude higher, showcasing their strong resistance to interference.^[^ ^234^ ^]^

### 3.6 Potential Future AM Technologies for OECTs

Additive manufacturing methods such as inkjet, aerosol‐jet, and screen printing provide the scalability and cost‐effectiveness necessary for large‐area device production. This shift toward additive techniques is essential for the widespread adoption of OECTs in practical applications. An overview of OECTs fabricated with fully‐additive techniques is provided in **Table** **2**, considering the materials, geometries, on/off current ratio, and transconductance.

**Table 2:** Overview of OECTs fabricated with fully‐additive techniques.

Channel material	G electrode material	S/D electrode material	Fabrication method	Structure	Channel length [µm]	Electrolyte	g_m_ [mS]	*I*_on_/*I*_off_	Refs.
PEDOT:PSS	PEDOT:PSS	Carbon	Screen printing	Planar, top gate	100	polyDDA + H2O		10^5^	[37]
PEDOT:PSS	PEDOT:PSS	Carbon	Screen printing	Planar, top gate	150	polyDDA + H2O	0.7	10^4^	[38]
PEDOT:PSS	PEDOT:PSS	Silver	Aerosoljet printing	Planar, side gate	200	NaCl	0.5	10	[41]
PEDOT:PSS	PEDOT:PSS	GO/CNT	Dispensing	Planar, side gate	77	PSS:Na + NaCl	7	2.5 10^3^	[43]
PEDOT:PSS	Ag/AgCl	Carbon	Dispensing + Direct Write	Planar, side gate	400	PSS:Na + NaCl	0.75	10^3^	[44]
PEDOT:PSS	PEDOT:PSS	Carbon	Screen printing	Planar, top gate	100	AFIVV009		10^4^	[20]
PEDOT:PSS	PEDOT:PSS	Carbon	Screen printing + Aerosoljet printing	Planar, top gate	70	E003	1.1	10^4^	[54]
P(g42T‐T)	Ag/AgCl	Carbon	Screen printing + Spray coating	Planar, side gate	200	PQ‐10 + NaCl	0.17	10^3^	[22]
BBL	Ag/AgCl	Carbon	Screen printing + Spray coating	Planar, side gate	200	PSS:Na + NaCl	0.18	2 10^3^	[22]
pgBTTT	PEDOT:PSS	Carbon	Screen printing + Inkjet printing	Planar, top gate	80	E003	0.6	10^4^	[66]
PEDOT:PSS	Ag/AgCl	Silver	Inkjet printing	Planar, pellet gate	32	PBS	15		[39]
PEDOT:PSS	Ag/AgCl	Silver	Dispensing	Planar, side gate	1500	PVA + NaCl	1.2	1.5 10^2^	[93]
PEDOT:PSS	PEDOT:PSS	Carbon	Screen printing	Planar, top gate	200	AFIVV009		10^4^	[97]
PEDOT:PSS	PEDOT:PSS	PEDOT:PSS	Inkjet printing	Planar, side gate		PBS			[235]
PEDOT:PSS	PEDOT:PSS	Carbon	Screen printing	Planar, top gate	80	AFIVV009	1	10^4^	[95]
PEDOT:PSS	Ag/AgCl	Carbon	3D printing + Inkjet printing	Planar, pellet gate	3000	PBS	0.2	10	[6]
PEDOT:PSS	Ag/AgCl	Silver	Dispensing	Planar, side gate	118	PVDF‐HPF + EMIM:TFSI	1.5	10^4^	[68]
PEDOT:PSS	Ag/AgCl	Silver	Dispensing	Vertical, side gate	2	PVDF‐HPF + EMIM:TFSI	50	6 10^3^	[68]
PEGDA:PEDOT	Ag/AgCl	PEGDA:PEDOT	Stereolithograpy	Planar, pellet gate	2000	NaCl	2.5	3 10^3^	[236]
PEDOT:PSS	Carbon	Carbon	Screen printing	Planar, side gate	2000	PBS	0.7		[237]
PEDOT:PSS	PEDOT:PSS	Carbon	Screen printing + Laser ablation	Planar, top gate	25	E009		10^3^	[98]
PEDOT:PSS	PEDOT:PSS	Carbon	Screen printing + Inkjet printing	Planar, side gate	3000	NaCl	0.38		[69]
PEDOT:PSS	Ag/AgCl	Silver	Dispensing	Planar, pellet gate	694	NaCl	31.8	10^3^	[231]

In recent years printed electronics have revolutionized the fabrication of large‐scale, low‐cost electronic systems, opening avenues for novel applications across various fields. Recent advancements, particularly in miniaturization, have significantly reduced the gap between printing and lithographic processes. Cutting‐edge technologies such as e‐jet printing,^[^ ^50^ ^]^ ultra‐precise dispensing (UPD),^[^ ^127^ ^]^ electromigration‐induced break junctions (EIBJ),^[^ ^238^ ^]^ reverse‐offset printing,^[^ ^239^ ^]^ and nanoimprint lithography (NIL)^[^ ^240^ ^]^ enabled the creation of sub‐micrometer features, redefining precision in printed electronics.

Electrohydrodynamic (EHD) printing leverages electric fields instead of conventional thermal or acoustic forces to achieve finer resolutions. In contrast to the conventional inkjet technique, by manipulating factors like voltage intensity and nozzle‐substrate spacing, EHD printing can produce droplets and micro/nanowires smaller than nozzle diameters.^[^ ^241^ ^]^ This method supports high‐viscosity inks, with advanced techniques like tip‐assisted EHD enhancing resolution without requiring extremely narrow nozzles or excessively high voltages.

Ultra‐precise dispensing (UPD) offers remarkable flexibility, enabling direct, maskless deposition of high‐viscosity materials on various substrates, including flexible and pre‐patterned surfaces. UPD's precision stems from controlled micro‐ and nano‐sized nozzle movement and pressure application,^[^ ^127^ ^]^ capable of forming complex shapes and vertical interconnections.^[^ ^242^ ^]^ Its nanocapillary variant further pushes the boundaries of precision by utilizing glass pipettes and feedback systems akin to atomic force microscopy. The pipette can be seen as a nano‐fountain pen printing any kind of ink on any substrate.^[^ ^243^ ^]^

Electromigration‐induced break junctions (EIBJ) exploit the mass transport generated by the migration of ions in metals to fabricate reliable nanogaps, with widths ranging from 1 to 30 nm. This method ensures high throughput and reproducibility, making it an efficient choice for nanoscale applications.^[^ ^238^, ^244^ ^]^

Reverse‐offset printing refines gravure printing by transferring coated semi‐dried thin ink films from low‐surface‐energy blankets to substrates. The blanket is impressed onto a cliché with intaglio patterns, and the impression results in adhesion between the ink film and the top surface of the cliché. At the same time, the blanket produces a bulge that penetrates into the intaglio pattern. This bulge produces a concentration of shear stress at the edges of the ink film. Because of the softness of the blanket, a bulge occurs and the ink film also deforms along the bulge. Finally, the impression is removed and the blanket is released from the cliché.^[^ ^239^, ^245^, ^246^ ^]^

Nanoimprint lithography (NIL) has been widely used to achieve high‐resolution patterning and can be classified as either a thermal or ultraviolet (UV) process. In a typical thermal NIL process, a mold is first heated above the glass transition temperature of the thermoplastic polymer resist. As the heated mold is compressed against the resist, the resist heats up and the pattern on the mold can be fully transferred onto the resist. NIL is a subtractive patterning process consisting of several steps and is not in itself a printing technique. Nevertheless, it is considered a good candidate for high‐resolution patterning in roll‐to‐roll manufacturing processes.^[^ ^247^, ^248^, ^249^ ^]^

While these advancements promise unprecedented precision and versatility, they also introduce challenges, including for example costs, complexity limitations in compatible inks, substrates, and rapid prototyping. Nevertheless, they present significant potential for the future generation of fully printed OECTs, widening the palette of OECT printing methods (see **Table** **3**).

**Table 3:** Current and future technologies for OECT fabrication. * These values correspond to routinely achievable values. Parallelization refers to the ability to process multiple devices simultaneously, enhancing efficiency and throughput. Mask/Mold highlights whether a mask or mold is required during the process. Automatization denotes the extent to which the process can be fully automated, reducing manual intervention. Design integration assesses the capability to incorporate all device components. For instance, Low: Integration of the gate electrode is not feasible; Medium: Integration of a non‐polarizable gate electrode is possible; High: Both polarizable and non‐polarizable gate electrodes can be easily integrated. Design flexibility measures the ease and speed of modifying designs, such as the ability to update designs via CAD software or the necessity of creating new masks or molds. Complexity evaluates various parameters, including the number of process steps, the difficulty of adding or removing materials, and the ease of substituting process materials. Substrate coating refers to whether the entire substrate needs to be coated to achieve specific features. Finally, OECT indicates whether the technology has been already utilized for OECT fabrication.

Printing method	Ink viscosity (cP)	Velocity [m ^−1^s]^*^	Line width / space [µm]^*^	Parallelization	Mask / Mold	Automatization	Design integration	Design flexibility	Complexity	Substrate coating	OECT
Inkjet	1 – 100	0.01 – 0.1	30 – 50	✗	✗	✗	High	Medium	Low	✗	✓
E‐jet	1 – 10^4^	0.01 – 0.1	1	✗	✗	✗	Medium	High	High	✗	✗
Offset	100 – 10^5^	10	10	✓	✓	✓	Medium	Low	High	✗	✗
Gravure	100 – 10^3^	0.15 – 15	10 – 50	✓	✓	✓	Medium	Low	Medium	✗	✓
Screen	500 – 10^5^	0.1 – 1	30 – 50	✓	✓	✗	High	Low	Low	✗	✓
Reverse‐offset	1 – 5	slow	1 – 10	✓	✓	✓	Medium	Low	High	✗	✗
Transfer	‐	slow	0.1	✓	✓	✗	Medium	Low	Medium	✗	✓
Aerosol jet	1 – 50	0.1 – 1	50 – 150	✗	✗	✗	High	High	Medium	✗	✓
Extrusion	500 – 105	0.01 – 0.15	100 – 250	✗	✗	✗	High	High	Low	✗	✓
Spray	1 – 50	‐	50 – 150	✓	✓	✗	High	Low	Low	✗	✓
UPD	5 – 10^5^	slow	1 – 250	✗	✗	✗	High	Medium	Low	✗	✗
Nanoimprint	‐	slow	0.01	✓	✓	✗	Medium	Low	High	✗	✓
Photolitography (2–layer peel‐off)	1 – 200	slow	1 – 10	✓	✓	✗	Medium	Low	High	✓	✓
Photolitography (photoresist)	1 – 200	slow	10	✓	✓	✗	Medium	Low	High	✓	✓

## 4 Emerging Applications

### 4.1 Chemical Sensing and Biosensors

**Figure 10:**
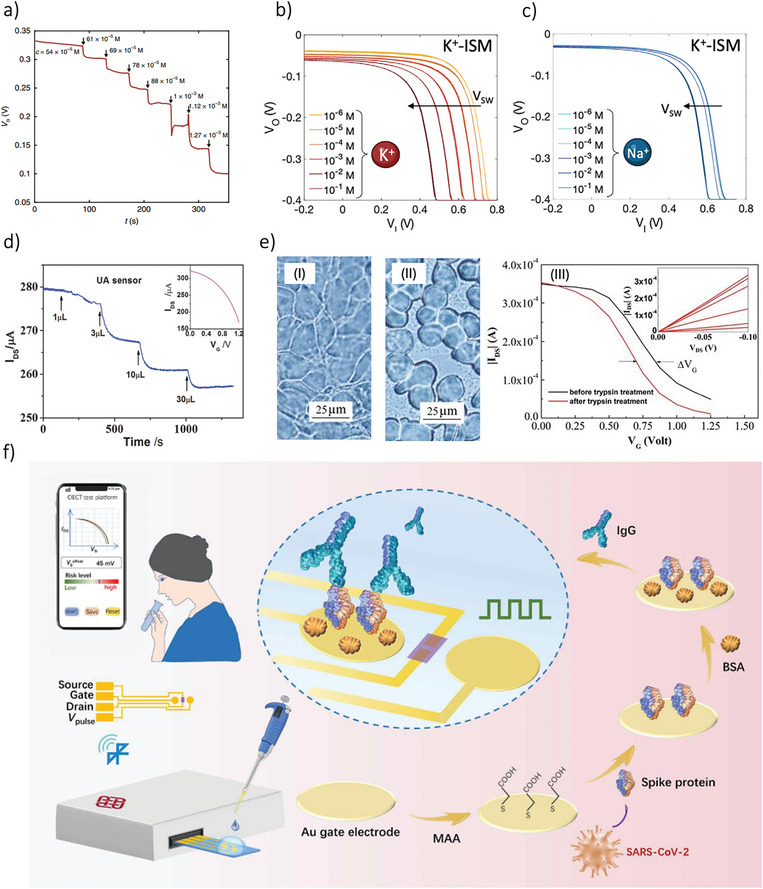
a) Real‐time high‐sensitivity ion detection, performed through a complementary inverter, made with a PEDOT:PSS p‐type OECT and a BBL n‐type OECT. Adapted with permissions.^[^ ^23^ ^]^ Copyright 2020, Springer Nature. b‐c) Measured input‐output characteristics of ten consecutive measurements at various K**^+^** (b) and Na**^+^** (c) concentrations in a current‐driven configuration ion‐selective OECT. Adapted with permissions.^[^ ^234^ ^]^ Copyright 2024, Wiley‐VCH. d) Channel current responses of a Uric Acid‐sensitive OECT with a UOx‐GO/PANI/Nafion‐graphene/Pt gate electrode characterized in PBS solution before and after the additions of saliva of different volumes. Inset: transfer characteristic of the OECT measured in PBS solution. Adapted with permissions.^[^ ^269^ ^]^ Copyright 2014, Wiley‐VCH. e) (I)(II) Optical images of cancer cells cultured on PEDOT:PSS films before and after being treated with trypsin solution. (III) *I*_DS_ versus *V*_G_ (transfer characteristics) of an OECT with cancer cells measured in the culture medium before and after being treated with trypsin solution. *V*_DS_  = −0.1 V. Inset: *I*_DS_ versus *V*_DS_ (output characteristics) of the device measured in the culture medium before and after being treated with trypsin solution. From top to bottom, the curves were measured at the *V*_G_ of 0, 0.25, 0.50, 0.75, 1.00, and 1.25 V, respectively. Adapted with permissions.^[^ ^270^ ^]^ Copyright 2010, Wiley‐VCH. f) Scheme of the portable sensing system and the gate modification processes of the IgG sensor. Adapted with permissions.^[^ ^57^ ^]^ Copyright 2021, AAAS.

OECTs have emerged as transformative components in advancing technologies across diverse applications, particularly in biosensing. Their unique capability to interact seamlessly with electrochemical environments has catalyzed the development of sophisticated chemical and physical sensors, enabling real‐time monitoring of critical biological indicators such as pressure, temperature, touch, etc.^[^ ^67^, ^138^, ^250^, ^251^, ^252^, ^253^, ^254^ ^]^ In chemical sensing, OECTs have redefined the detection of ions (e.g., Ca^2^⁺, K⁺, Cl⁻) and gases (e.g., NO, NO₂, H₂S), essential to physiological processes.^[^ ^86^, ^255^, ^256^, ^257^, ^258^ ^]^ These devices translate concentrations of chemical substances into precise electrical signals, mimicking the sensory functions of the human body. A notable example is the continuous NO gas sensor, which exhibits a broad detection range (3nM–100 µM) and exceptional sensitivity (94 mV dec⁻¹), enabling advancements in therapeutic and diagnostic applications.^[^ ^256^ ^]^ Similarly, miniaturized and flexible ion‐selective OECT systems have been developed for multiplexed ion sensing, enhancing environmental and biomedical analyses (**Figure** **10**a–c).^[^ ^23^, ^234^ ^]^ These innovations provide invaluable insights into chemical processes, advancing our understanding of health and environmental systems.^[^ ^257^ ^]^ OECTs have demonstrated not only an excellent performance for invasive and noninvasive measurements such as ECG, EMG, and EEG,^[^ ^14^, ^19^, ^259^ ^]^ but have also revolutionized biosensing by enabling the detection and monitoring of biomarkers, which serve as indicators of disease or biological processes. OECTs have been successfully used as many types of sensors (Figure 10d–e), including lactate,^[^ ^260^ ^]^ glucose,^[^ ^261^ ^]^ dopamine,^[^ ^262^ ^]^ DNA,^[^ ^263^ ^]^ bacteria,^[^ ^264^ ^]^ and protein.^[^ ^265^ ^]^ These devices utilize specific biosensitive materials such as enzymes, metabolites, and antibodies to translate biological substance concentrations into electrical signals with remarkable accuracy.^[^ ^14^, ^16^, ^57^, ^259^, ^266^, ^267^, ^268^ ^]^ For example, during the COVID‐19 pandemic, miniaturized OECT platforms for SARS‐CoV‐2 IgG detection demonstrated rapid, efficient, and portable diagnostic capabilities. By employing gate functionalization and enhancing antigen‐antibody binding through electrical pulses, these devices addressed the limitations of traditional diagnostic tools, offering a streamlined approach to disease detection and monitoring (Figure 10f).^[^ ^57^ ^]^

### 4.2 Neuromorphic

**Figure 11:**
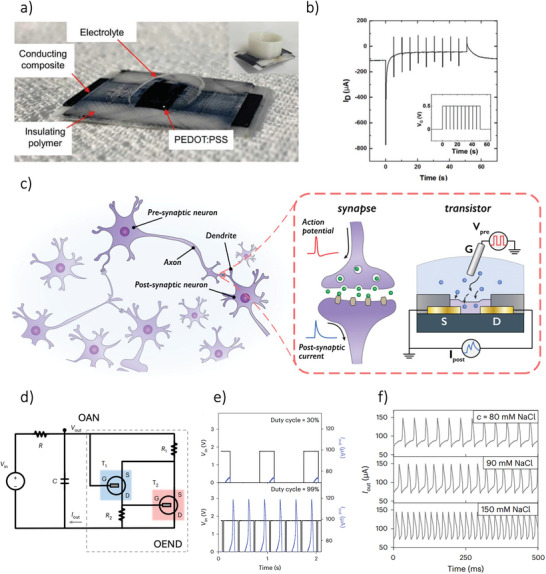
a) Hybrid 3D/inkjet printed neuromorphic OECT, and b) fast adaptation response of the drain current, measured by applying a train of gate voltage pulses. Adapted with permissions.^[^ ^6^ ^]^ Copyright 2020, Wiley‐VCH. c) Schematic analogy of synapse and OECT operation. Adapted with permissions.^[^ ^5^ ^]^ Copyright 2021, Springer Nature. d) Circuit diagram of the organic electrochemical non‐linear device (OEND), as a part of the OAN. T1 is the normally on OECT, T2 is the normally off OECT, and R1 and R2 are the two resistors of the OEND. e) In‐liquid temporal integration (100 mM NaCl). I_OUT_ current firing occurs as a response to short interstimulus time intervals or high‐duty cycles in the input voltage *V*_IN_. And f) Representative waveforms of the OAN firing response for different ionic concentrations (aqueous NaCl). Adapted with permissions.^[^ ^4^ ^]^ Copyright 2022, Springer Nature.

OECTs have shown immense promise in replicating the behavior of biological synapses and neurons, a breakthrough for neuromorphic computing and brain‐inspired systems. Their ability to modulate electrical and ionic signals mirrors the complex signal processing of biological systems, paving the way for advances in artificial intelligence and adaptive computing.^[^ ^3^, ^4^, ^5^, ^169^, ^259^ ^]^ This was explored by Mangoma et al., who integrated cost‐effective 3D and inkjet technologies to produce fully printed neuromorphic OECTs, (**Figure** **11**a)^[^ ^6^ ^]^ These devices exhibited depletion‐mode operation, paired‐pulse depression behavior, and evidence of adaptation to support the translation to neuromorphic devices. By applying different pulse durations to the gate electrode, they demonstrated the intrinsic memory response of the OECTs and its rapid adaptation to new steady states, (Figure 11b). In recent years, various research groups have focused on the neuromorphic behavior of OECTs. For example, Doremaele et al. demonstrated electrochemical random‐access memories (EC‐RAMs), leveraging the neuromorphic properties of OECTs to perform complex classification tasks directly in hardware. This innovation holds promise for applications in wearable, implantable, and point‐of‐care devices.^[^ ^271^ ^]^ Recently, Ji et al., showed associative learning using synaptic OECTs. Their work is based on the analogue operation between the OECT gate voltage and channel current, with the action potentials created by the release of neurotransmitters (Figure 11c), and simulating synaptic functions like short‐term plasticity (STP), long‐term plasticity (LTP), and spiking time‐dependent plasticity (STDP). The poly(3,4‐ethylenedioxythiophene):tosylate (PEDOT:Tos)/ Polytetrahydrofuran (PTHF) ‐based OECTs produced in their synaptic device can associate two physical inputs (light and pressure) by integrating a pressure sensor and a photoresistor with a volatile and a non‐volatile OECT.^[^ ^5^ ^]^

Recently, Sarkar et al. presented an OECT‐based organic artificial neuron (OAN) for in‐liquid neuromorphic sensing and bio‐interfacing, that combines two p‐type OECT, one in depletion mode and one in accumulation mode, in a compact organic electrochemical non‐linear device (OEND) that exhibits negative differential resistance. The OAN responds to ionic species commonly found in the extracellular space, and its spiking response is sensitive to typical physiological and pathological ionic concentration ranges (5–150 mM). Small‐amplitude (1–150 mV) electrochemical oscillations and noise in the electrolytic medium shape the neuronal firing properties (Figure 11d–f).^[^ ^4^ ^]^ Subsequently, by combining experiments with numerical simulations, Belleri et al., have designed and explained the wide biorealistic repertoire of organic electrochemical artificial neurons including their firing properties, neuronal excitability, wetware operation, and biohybrid formation. They unraveled the operation of organic electrochemical artificial neurons by the investigation of the OEND, and focusing on the OAN fundamental operations such as spiking frequency, voltage, and current amplitude of the output oscillations, power consumption, and energy per spike, paving the way for a model‐based design of OAN.^[^ ^3^ ^]^

Furthermore, OECTs allowed biorealistic emulation of neuronal behavior through the use of anti‐ambipolar materials, such as BBL. Harikesh et al. demonstrated the capability of these materials to emulate the activation and inactivation dynamics of sodium channels in neurons. By leveraging the ion tunability of BBL, these OECTs replicate neural spiking at a bioplausible frequency of 100 Hz, showcasing their potential for creating sophisticated neuromorphic systems that mimic the behavior of biological neural networks.^[^ ^169^ ^]^

### 4.3 Transient and Green Electronics

**Figure 12:**
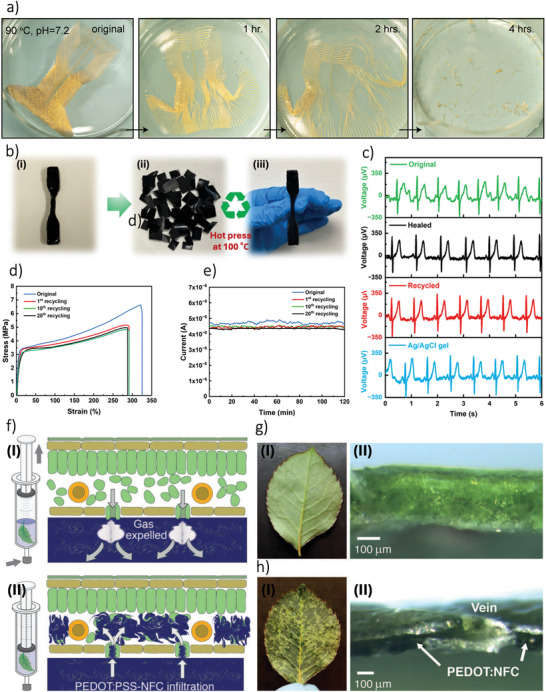
a) Photographs of devices collected at various times following immersion in 1X PBS (pH 7.2) solution at an elevated temperature (90 °C) for accelerated degradation. Adapted with permissions.^[^ ^103^ ^]^ Copyright 2023, Wiley‐VCH. b) Photographs of the original PEDOT: PSS/PU/PEG sample i) after being cut into small pieces ii) and after remolding by hot‐pressing iii). c) ECG signals recorded using PEDOT:PSS/PU/PEG film electrodes before (green) and after self‐healing (black) and recycling (red) and commercial Ag/AgCl gel (blue) electrodes. d) Comparison of tensile stress–strain curves for the sample before and after recycling (1, 10, and 20 times), and e) current versus time plot before and after recycling (1, 10 and 20 times) (applied voltage of 0.2 V). Adapted with permissions.^[^ ^65^ ^]^ Copyright 2024, Royal Society of Chemistry. And PEDOT‐infused leaves in f) (I) Vacuum infiltration. Leaf placed in PEDOT:PSS–NFC solution in a syringe with air removed. The syringe is pulled up, creating negative pressure and causing the gas inside the spongy mesophyll to be expelled. (II) When the syringe returns to standard pressure, PEDOT: PSS–NFC is infused through the stomata, filling the spongy mesophyll between the veins. g) Photograph of the bottom (I) and cross section (II) of a pristine rose leaf before infiltration. h) Photograph of the bottom (I) and cross section (II) of the leaf after PEDOT:PSS–NFC infusion. Adapted with permissions.^[^ ^273^ ^]^ Copyright 2015, AAAS.

The development of transient and green electronics aligns with the growing emphasis on sustainability. OECTs made from bioresorbable, biodegradable, and eco‐friendly materials are being explored for a large palette of application fields, including for example temporary medical implants, environmentally conscious devices, and circular economy in electronics.^[^ ^65^, ^66^, ^103^, ^201^, ^272^ ^]^ Printing methods, such as inkjet and aerosol‐jet printing, offer unmatched flexibility, precision, and scalability, making them ideal approaches for producing biodegradable and biointegrable OECT‐based electronics.^[^ ^69^, ^71^, ^133^, ^273^, ^274^ ^]^ In recent years, researchers focused on biodegradable OECTs have made significant progress, starting with the development of devices on biodegradable substrates. Several experimental studies have successfully produced OECTs on eco‐friendly materials such as PLA,^[^ ^173^ ^]^ cellulose acetate (CA),^[^ ^99^ ^]^ and cellulose diacetate (DCA).^[^ ^44^ ^]^ These environmentally sustainable OECTs hold great potential for use as disposable biochemical sensors. An illustrative example is the fully inkjet‐printed disposable glucose sensor developed by Bihar et al.^[^ ^70^ ^]^ This innovative device utilizes all‐PEDOT:PSS biosensors to accurately measure physiologically relevant glucose levels in human saliva. The detection mechanism relies on enzymatic electrochemical sensing, achieved by integrating the enzyme glucose oxidase with an electron mediator as the biorecognition element. Fabricated on readily available commercial paper substrates, these sensors exhibit an operational range spanning from 0.025 mM to 0.9 mM. The system demonstrates high sensitivity, making it well‐suited for identifying abnormal glucose concentrations in saliva.^[^ ^70^ ^]^ Another significant example of OECT‐based transient electronics was demonstrated by Wu et al., who developed an array of 100 bioresorbable OECTs designed for in vivo spatiotemporal mapping of brain activity.^[^ ^103^ ^]^ Their study highlighted the remarkable capabilities of these implanted transient devices: i) they obtain uniform and high transconductance of up to 9.0 mS in vitro, enabling enhanced electrophysiological (EP) signal recording with a signal‐to‐noise ratio (SNR) of up to 37 dB in vivo; ii) they operate across 100 channels to collect EP signals at the cellular level in a rat module; iii) they distinguish between normal and pathological regions within the animal model during active in vivo recordings; and iv) the spontaneous devices degradation after completing their function, eliminating the need for secondary removal surgery. As shown in **Figure** **12**a, the devices dissolve in PBS, they are composed by utilizing a biodegradable poly(lactic‐co‐glycolic acid) (PLGA) substrate, Au electrodes, a water‐soluble PVA support substrate, and the organic semiconductor PEDOT:PSS.

To enhance green electronics, other research groups focused their studies on innovative materials and devices, which can be eco‐friendly and recyclable. Examples include the substitution of the commonly used harmful solvents such as chloroform, with the dihydrolevoglucosenone (Cyrene), a non‐toxic, cellulose‐derived, and biodegradable solvent for the pgBTTT channel formulation of all‐printed accumulation‐mode OECTs^[^ ^66^ ^]^ and the eco‐friendly screen printing of silver nanowires by introducing biodegradable binder and a green solvent with no toxic surfactants in the ink formulation.^[^ ^272^ ^]^ Another notable example is provided by Kim et al., they have fabricated a self‐healing, stretchable (elongation at break of ∼350%, toughness of ∼24.6 MJ m^−3^, and high Young's modulus of ≈50 MPa) and recyclable polyurethane‐PEDOT:PSS conductive blends, contributing to sustainable electronics by replacing critical materials in targeted applications, such as sensing, bioelectronics, wearable electronics, and soft robotics. Particularly, they show that in PEDOT:PSS/PU/PEG material there was no significant deterioration in the electrical properties even after 20 remolding cycles (Figure 12b‐e).^[^ ^65^ ^]^

Interestingly, recent works use the direct integration of the OECTs in living structures and substrates. Berggren and co‐workers reported analog and digital organic electronic circuits and devices manufactured in living plants. The four key components of a circuit have been achieved using the xylem, leaves, veins, and signals of the plant as the template and integral part of the circuit elements and functions. They use cuttings of rosa floribunda (garden rose) as a model plant system and a PEDOT‐S:H xylem wire that simultaneously served as the transistor channel, source, and drain of an OECT with on/off ratio of ≈40, a transconductance of 14 µS. Further connecting various OECTs they obtain a complex xylem‐templated circuits, namely, xylem logic with NOR logic behavior. Finally a PEDOT:PSS–NFC‐based leaf OECD was created using vacuum infiltration (Figure 12f–h).^[^ ^273^ ^]^ Recently, Tran et al. developed a PEDOT:PSS‐based wood OECT (WOECT) with an electrical conductivity of up to 69 Sm^−1^ and capable of modulating an electrical current in a porous and thick transistor channel (1 mm) with an on/off ratio of 50. The WOECTs are simply produced by coating the wood with the polymer and assembling the three pieces of conductive wood to form the channel and the double gate electrodes.^[^ ^274^ ^]^

### 4.4 Self Healable and 4D Electronics

**Figure 13:**
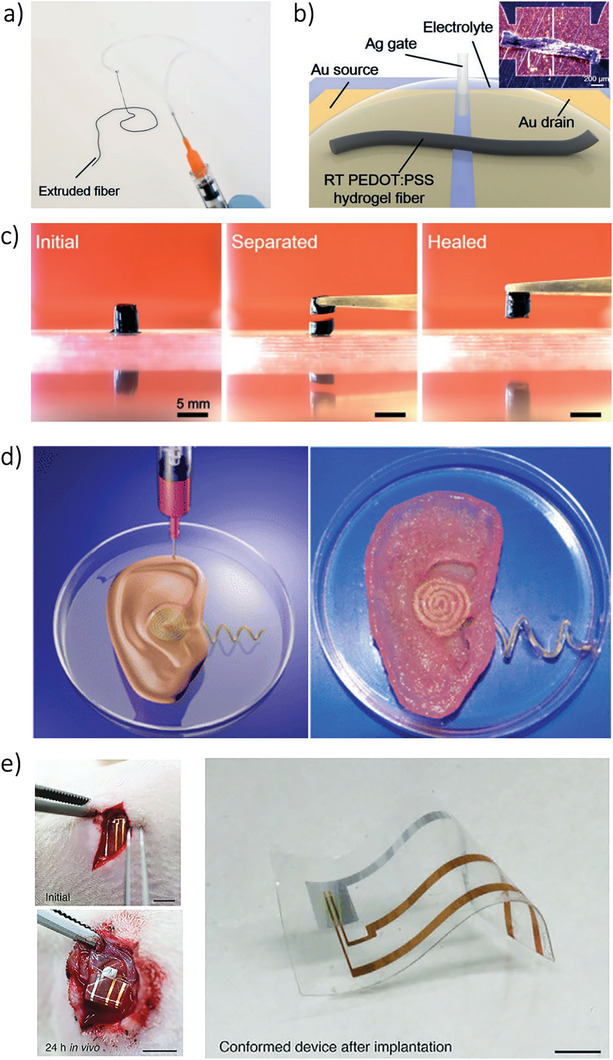
a) Extruded RT‐PEDOT:PSS hydrogel fibers with a syringe. b) Schematic of the fabricated OECTs with injected RT‐PEDOT:PSS hydrogel fiber. The hydrogel fiber was freeze‐dried after printing on the Au electrodes which have a gap of 10 µm; the inset shows the real optical image of the freeze‐dried fiber on the source–drain electrodes. c) Mechanical healing of the RT‐PEDOT:PSS hydrogel by placing two separated RT‐PEDOT:PSS hydrogels (mildly dehydrated) together for 5 min. Adapted with permissions.^[^ ^64^ ^]^ Copyright 2019, Wiley‐VCH. d) Illustration of the extrusion printing phase of the bionic ear and image of the printed bionic ear immediately after printing. Adapted with permissions.^[^ ^275^ ^]^ Copyright 2013, American Chemical Society. e) An OTFT coated with SMP conforms to the body tissue of a living rat after being implanted for 24 h. The scale bars are 5 mm for the left two images and 2 mm for the right, large image. Adapted with permissions.^[^ ^288^ ^]^ Copyright 2014, Wiley‐VCH.

Another research direction in the field of OECTs relates to the mechanical stability of the devices over time and the ability to resist wear and tear. To enhance the practicality of employing OECTs in smart and wearable devices, it is also desirable to look at active materials with good flexibility and durability to withstand wear, scratching, and unexpected damage from mechanical bending. The ability of electronic materials to self‐heal autonomously like living tissues is an interesting approach to fabricate devices with high reliability and longer lifetime. One of the first approaches to self‐healing active materials was made by Zhang et al. who mixed PEDOT:PSS with 4‐dodecylbenzenesulfonic acid (DBSA). The demonstrated the unique room‐temperature gelling ability of PEDOT:PSS directly extrudable from a syringe (injectable)^[^ ^64^ ^]^ can find potential applications in minimally invasive therapeutics such as nerve regeneration and brain stimulation without causing any trauma. As shown in **Figure** **13**a–c they have demonstrated the extrusion of channel material of an OECT with self‐healable electrical and mechanical characteristics. Other examples are the first reported all‐solid state OECT with a self‐healable channel deposited by spray coating,^[^ ^62^ ^]^ and the PEDOT:PSS/PU/PEG material produced by Kim et al.^[^ ^65^ ^]^ The ability of materials to change over time by “self‐healing” paves the way for the advent of intelligent materials, which not only actively react to stimuli, but can also be programmed. As additive manufacturing techniques are able to create highly integrated 3D multifunctional constructs, many researchers are exploring these emerging technologies to fabricate geometrically complex and biocompatible devices and scaffolds that have electrical functionalities. For example, 3D printed a pair of left and right bionic ears that are capable of enhanced auditory sensing for radio frequency reception and stereo audio music listening have been recently demonstrated by Mannoor et al. (Figure 13d).^[^ ^275^ ^]^ 4D printing includes the material utilization that can alter their shapes/ properties with time. It allows for objects to be designed to react to certain stimuli and modify their shape accordingly, it involves the phenomena of shape programming that refers to the design and programming of structures or materials to alter their configuration or shape in response to some specific stimuli. The key difference between these printing technologies is that 4D includes the material usage that can self‐transform, while 3D is limited to create static objects layer by layer.^[^ ^276^ ^]^ The term “4D‐printing” was first conceived in the last decade^[^ ^277^ ^]^ and since then, it has grown rapidly and researchers exploring the use of various techniques and materials to build objects that can alter their texture, function, and shape over time. 4D printing is actually used for printing functional electronics,^[^ ^278^ ^]^ antennas,^[^ ^279^ ^]^ self‐morphing circuits,^[^ ^280^ ^]^ and sensors;^[^ ^281^ ^]^ through the use of smart materials such as shape memory polymers (SMPs) and elastomers,^[^ ^282^ ^]^ hydrogels and aerogels,^[^ ^283^ ^]^ shape memory ceramics (SMCs),^[^ ^284^, ^285^ ^]^ composites, and nanomaterials.^[^ ^286^, ^287^ ^]^ For example, Reeder and co‐workers^[^ ^288^ ^]^ reported a change of a thiol‐ene/acylate‐based organic transistor after implanting it into a living tissue after 24 h (Figure 13e), and a thiol‐ene/acrylate‐based nerve cuff was also reported by Ware et al.^[^ ^289^ ^]^ This nerve cuff was capable of recovering the original helix shape in vivo, while external heat was not required during the recovery process, which is essential for a biological device. The nerve cuff was reported to stimulate neural activity, including heart rate and oxygen saturation. Stiller and co‐workers^[^ ^290^ ^]^ reported an intracortical probe for neural recording and electrochemistry. The generated device was fully encapsulated in thiol‐ene/acylate polymer, and a 13‐week in vivo experiment was performed to assess the chronic intracortical recording and electrochemical stability of the device. In addition to these devices, Sekitani and co‐workers^[^ ^291^ ^]^ generated a flexible organic transistor. Additionally, also solution‐driven SMPCs have been demonstarted, for instance, Liang et al.^[^ ^292^ ^]^ developed a smart hydrogel with high stretchability, self‐healing property, and shape memory behavior. The hydrogel was made from the interpenetration of the network structure comprising polyacrylamide and phenylboronic acid grafted alginate, and its shape memory behavior was characterized by dehydration and rehydration. Showing the great potential of this technology and new OECT‐based fully printed electronics and bioelectronics.

## 5 Summary and Outlook

OECTs have established themselves as a pivotal technology in the realm of bioelectronics, environmental sensing, and flexible electronics. This review has underscored the critical interplay between fabrication techniques and device performance, highlighting the advantages of innovative additive manufacturing technologies. Despite the promising capabilities of OECTs, several challenges need to be addressed to fully realize their potential, and critical open challenges can be summarized as follows.

- *Geometrical precision and film homogeneity*: Achieving consistent device dimensions and uniformity in film thickness is crucial for optimizing OECT performances. Variations can lead to significant discrepancies in electrical characteristics.^[^ ^172^ ^]^
- *Material brittleness*: Organic mixed conductors often exhibit brittleness, which limits their mechanical reliability and performance in flexible applications.^[^ ^293^ ^]^
- *Manufacturing limitations*: Current additive manufacturing techniques may not support the intricate architectures required for next‐generation OECTs, hindering innovation in device design.
- *Sustainability concerns*: The environmental impact of electronic waste continues to be a pressing issue. Developing biodegradable OECTs is essential for aligning with sustainability goals but presents its own set of challenges in material (e.g., solvents) selection and processing (e.g., process temperature).^[^ ^69^, ^71^ ^]^
- *Integration with smart materials*: The integration of OECTs with advanced materials that can self‐repair or adapt to environmental changes is still in its infancy. Achieving reliable performance with such materials remains a significant hurdle.^[^ ^62^, ^65^ ^]^

Possible strategies to overcome these challenges include:

- *Enhanced additive manufacturing techniques*: Adopting advanced printing technologies like nanoimprint lithography (NIL), e‐jet printing, and micro‐dispensing can significantly improve the accuracy and reproducibility of OECT fabrication. These methods allow for more controlled deposition of materials, enabling the production of devices with improved geometrical precision and film uniformity.^[^ ^202^ ^]^
- *Material innovation*: Developing new organic materials with enhanced flexibility and mechanical strength can mitigate the brittleness issue. Researchers can explore hybrid materials that combine the desirable properties of multiple compounds to achieve improved performance and durability.
- *Biodegradable substrates*: By using biodegradable materials for both the active layers and substrates, OECTs can reduce their environmental impact. Ongoing research in biocompatible materials will be critical to create effective biodegradable electronics.^[^ ^44^, ^173^ ^]^
- *Self‐healing and smart materials*: The integration of self‐healing materials can significantly enhance the reliability of OECTs. Research into shape memory polymers and hydrogels, which respond to external stimuli, will facilitate the development of adaptive electronic systems.
- *Characterization and modeling*: Improved characterization techniques and modeling approaches can provide insights into the complex behavior of OECTs, aiding in the optimization of device architectures and material properties.

A key avenue for future exploration is the emergence of 4D electronics. Unlike 3D printing, which is limited to creating static objects layer by layer,^[^ ^276^ ^]^ 4D additive manufacturing enables the design of dynamic structures capable of self‐transformation. This is achieved through shape programming, where materials or structures are designed to change their configuration in response to specific triggers such as heat, moisture, or light. The synergy between biodegradable OECTs and 4D additive manufacturing technologies is poised to revolutionize the future of sustainable, responsive, and intelligent systems.^[^ ^278^, ^279^, ^280^, ^281^ ^]^ This convergence combines the environmental benefits of biodegradable materials with the dynamic capabilities of 4D printing, where devices can adapt and respond to external stimuli.^[^ ^282^, ^283^, ^284^, ^285^, ^294^ ^]^ The ability to create devices that can change properties or shapes in response to external stimuli opens up exciting possibilities for applications in soft robotics, smart wearables, and adaptive medical devices. This dynamic adaptation can significantly enhance the functionality of OECTs, allowing for more responsive and intelligent systems.

Moreover, while current research in additive manufacturing technologies has primarily focused on TFTs and OTFTs,^[^ ^202^ ^]^ it is crucial to expand this knowledge base to encompass OECTs. Techniques such as e‐jet printing,^[^ ^50^ ^]^ ultra‐precise dispensing,^[^ ^127^ ^]^ electromigration‐induced break junction (EIBJ),^[^ ^238^ ^]^ reverse‐offset printing,^[^ ^239^ ^]^ and nanoimprint lithography (NIL)^[^ ^240^ ^]^ offer tremendous potential for refining the fabrication of OECTs. These advancements could facilitate precise control over device architectures and material properties, leading to the development of high‐performance, fully printed OECTs on flexible and biodegradable substrates. By advancing materials optimization and device engineering, researchers can create robust, self‐healing electronic materials that withstand mechanical stress, enhancing the longevity and reliability of OECTs in practical applications. Moreover, the exploration of smart materials, such as shape memory polymers, hydrogels, and nanocomposites, can further improve OECT performance. The development of self‐healing materials, as demonstrated by recent studies, blending PEDOT:PSS with novel additives, can significantly enhance the durability and adaptability of OECTs. These innovations not only contribute to the reliability of OECTs but also support the broader goal of creating sustainable and efficient electronic devices.

In conclusion, the future of additive manufacturing OECT technology is characterized by the integration of sustainability, adaptability, and advanced printing techniques. The continued evolution of OECTs, driven by innovative materials, processes, and engineering designs, promises to reshape the landscape of next‐generation electronics, bioelectronics, and neuromorphics. As researchers bridge the gap between advanced additive manufacturing methods and OECT technology, they will pave the way for the development of multifunctional, adaptive (bio)electronic systems that are both intelligent and sustainable. This holistic approach will drive innovation in emerging fields positioning OECTs at the forefront of neuromorphic edge‐sensing and bioelectronics, reshaping the landscape of next‐generation intelligent and sustainable technologies.

## Conflict of Interest

The authors declare no conflict of interest.
